# Supplementary material for: The Balance between Mono- and NEDD8-Chains Controlled by NEDP1 upon DNA Damage Is a Regulatory Module of the HSP70 ATPase Activity
Source: Cell Rep. 2019 Oct 1;29(1):212–224.e8. doi: 10.1016/j.celrep.2019.08.070 (PMC6899524; doi:10.1016/j.celrep.2019.08.070)
Supplement: Document S1. Figures S1–S7 [file mmc1.pdf]

Cell Reports, Volume *29*

Supplemental Information

The Balance between Mono- and NEDD8-Chains Controlled by NEDP1 upon DNA Damage Is a Regulatory Module of the HSP70 ATPase Activity

Aymeric P. Bailly, Aurelien Perrin, Marina Serrano-Macia, Chantal Maghames, Orsolya Leidecker, Helene Trauchessec, M.L. Martinez-Chantar, Anton Gartner, and Dimitris P. Xirodimas

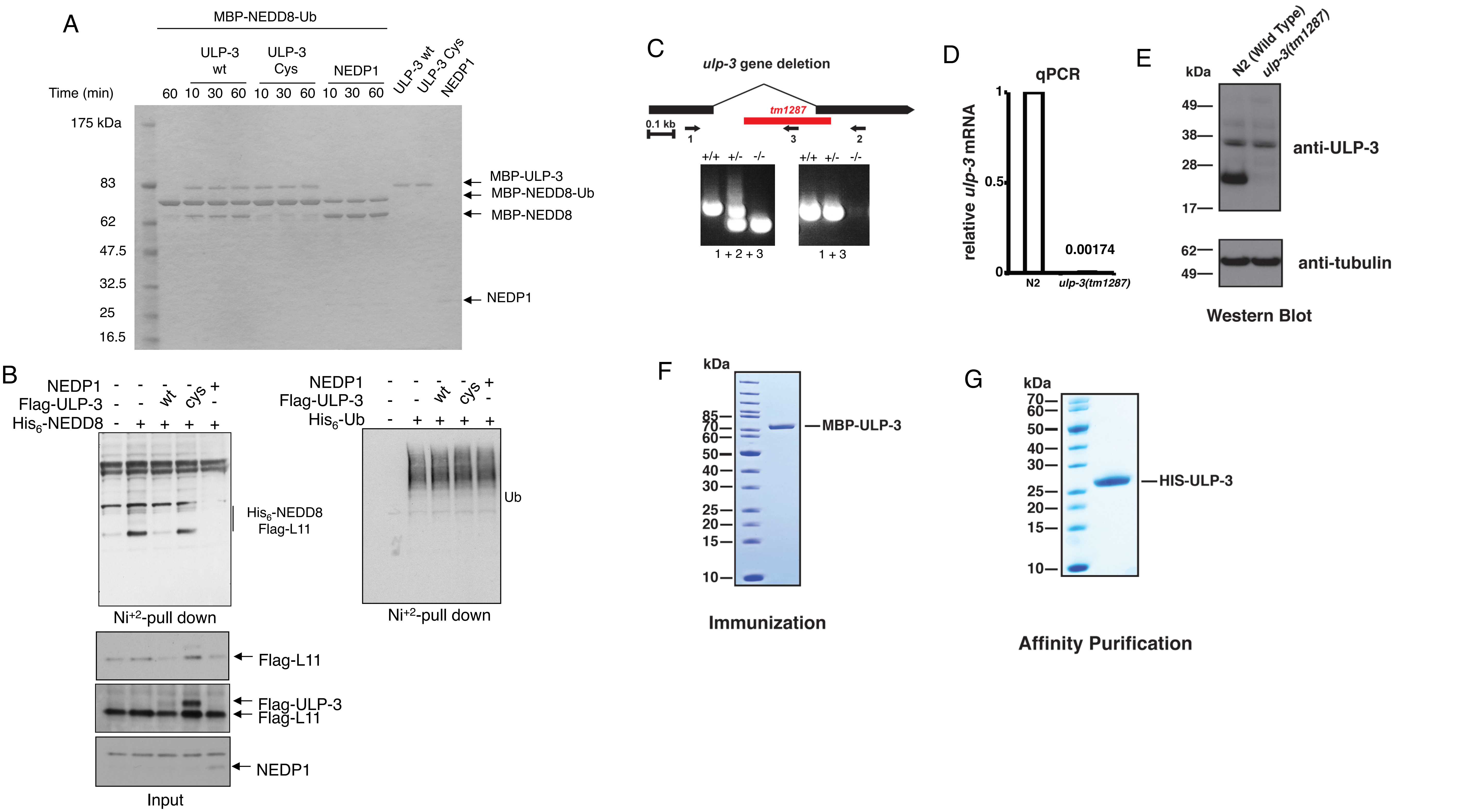

Figure S1. ULP-3 has NEDD8 processing and de-NEDDylating activity. The *ulp-3(tm1287)* allele abolishes *ulp-3* gene expression, related to Figure 1.

(A) Bacterially expressed wild type MBP-ULP-3 or Cys catalytic mutant were tested *in vitro* for NEDD8 processing activity using the MBP-NEDD8-Ub fusion. As control wild type bacterially expressed NEDP1 was used.

(B) H1299 cells were transfected with the indicated constructs. In all samples Flag-L11 was also co-transfected. 48hrs post-transfection His_6-NEDD8 conjugates were isolated with Nickel beads and western blot analysis was performed with anti-Flag antibody. Total cell extracts (input) were analysed with the indicated antibodies. Similar experiments were performed using His_6-ubiquitin and after Nickel purification ubiquitin conjugates were detected with anti-ubiquitin antibody (right panel).

(C) The *tm1287* allele structure demonstrated by the PCR products generated by the indicated primers. +/+, +/- and -/- indicate the N2/N2, N2/*ulp-3* and *ulp-3/ulp-3* genotypes respectively.

(D) The gene disruption is demonstrated by the *ulp-3* mRNA level measured by real time PCR. Relative expression levels were determined using γ-tubulin as standard (*tbg-1*). Fold induction was calculated as previously described (Bailly et al., 2010).

(E) Wild type and *ulp-3(tm1287)* mutant worms were synchronised and grown under standard conditions. Protein extraction was performed as described in Methods and the ULP-3 protein levels analysed by western blotting. α-tubulin was used as protein loading control.

(F) Recombinant MBP-ULP-3 protein, used for de-NEDDylation *in vitro* assays and rabbit immunisation (Supplemental information Fig. 1A), was produced in BL21 cells and purified with amylose resin as described in Methods. Purified MBP-ULP-3 protein was analysed by SDS-PAGE followed by Coomassie Blue staining.

(G)Recombinant His-ULP-3 (used to purify ULP-3 antibodies) was produced in BL21 cells and purified on a Cobalt column then analysed by SDS-PAGE followed by Coomassie Blue staining.

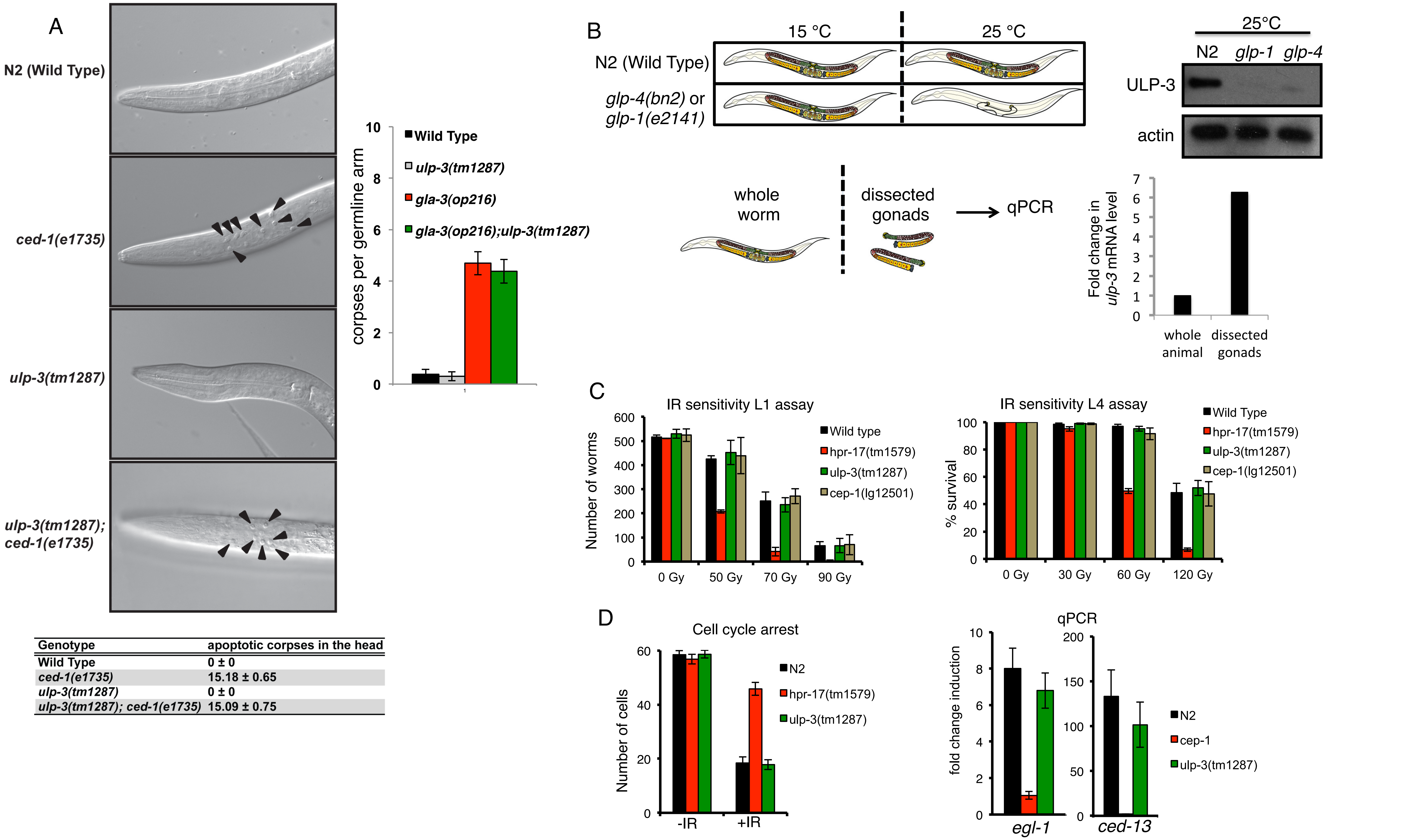

Genotype	apoptotic corpses in the head
Wild Type	0 ± 0
ced-1(e1735)	15.18 ± 0.65
ulp-3(tm1287)	0 ± 0
ulp-3(tm1287); ced-1(e1735)	15.09 ± 0.75

Figure S2. ULP-3 specifically affects the DNA damage-induced apoptotic pathway, related to Figure 1.

(A) Left panel: the apoptotic corpses that persist in the head until the L1 larvae stage in *ced-1(e1735)* animals were scored as in Bailly et al., 2010. Representative DIC pictures of the indicated genotype are shown. Average number of corpses, +/- SEM is represented (*n*=15) in the lower panel. In contrast to wild type, the *gla-3(op216)* mutant displays an elevated number of germ cell apoptosis without DNA damage referred as physiological apoptosis level. The double mutant *ulp-3(tm1287); gla-3(op216)* does not show a significant decrease in the number of apoptotic cells suggesting that ULP-3 does not regulate physiological germ cell apoptosis (right panel).

(B) Thermo-sensitive mutants glp-4(bn2ts) do not develop germ line tissue into adult gonads when exposed to restrictive temperature at early development stage, leading to animals essentially devoid of germ cell as depicted in the upper panel. Wild type and glp-4(bn2ts) synchronised worms were both switched to restrictive temperature at the L1 larvae stage (25°C). After reaching young adult stage, worm protein were extracted and ULP-3 protein level analysed by western blotting (upper right panel). RNA from whole animals or from dissected gonads from wild type animals was used for qPCR to monitor *ulp-3* expression. Values were normalised to tubulin expression and presented as fold change relative to whole animal *ulp-3* expression (lower right panel).

(C) The *ulp-3* deletion allele does not increase irradiation sensitivity following L1 (upper graph) or L4 (middle graph) larvae stage irradiation. Assays were performed as described (Bailly et al., 2010) using *hpr17* deletion mutant as a positive control (the RAD17 worm homologue).

(D) Left panel: *ulp-3* deletion does not impact on the mitotic cell cycle arrest triggered by the DNA damage checkpoint signalling pathway in *C. elegans* germ cells. Arrested cells were monitored and counted as previously described (Bailly et al., 2010). Right panel: Expression levels of 2 p53 regulated genes (egl-1 and ced-13) after IR (90Gy) in different genetic backgrounds. γ-tubulin expression was used to normalise values. Data represent the average (*n*=3) +/- SEM.

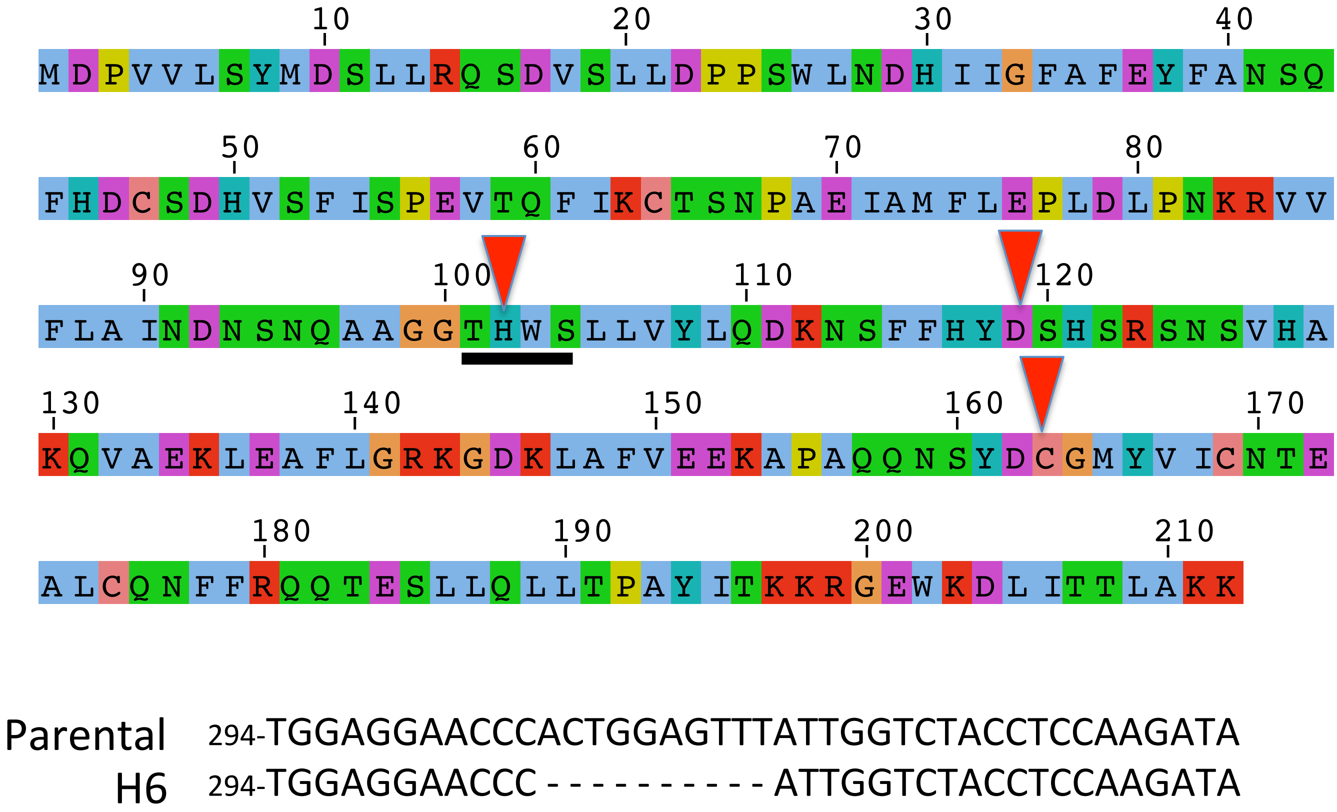

Figure S3. NEDP1 deletion by CRISPR/Cas9, related to Figure 3.
Sequencing of NEDP1 PCR products using genomic DNA as template from parental or H6 U2OS cells confirmed the indicated deletion of 10 base pairs in the NEDP1 gene creating an out of frame sequence and a stop codon 90 bases downstream. Red arrows indicate the position of the catalytic triad in NEDP1 and the black line the target area of the guide sequence at amino acid level.

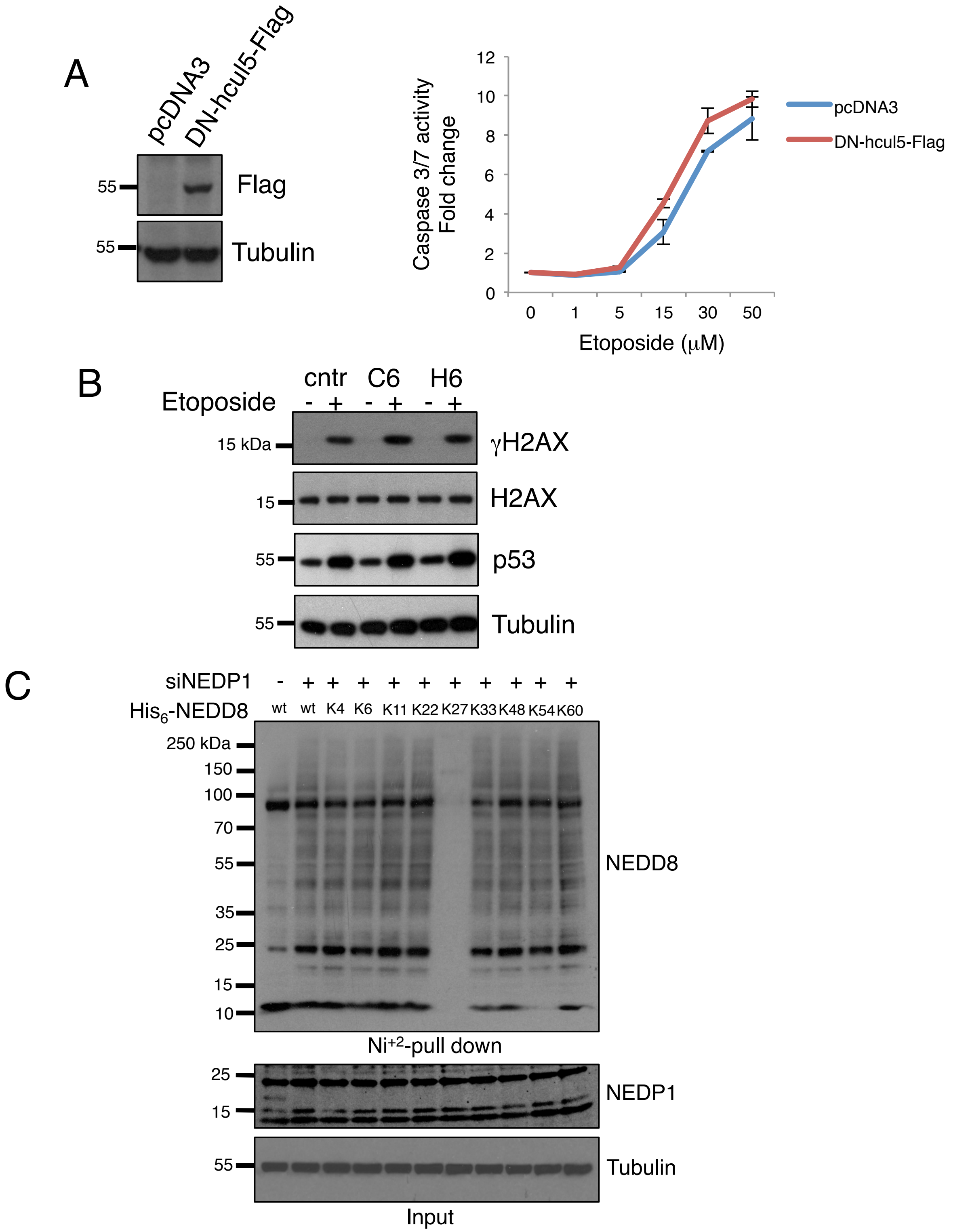

Figure S4. Characterisation of the DSBs-induced apoptosis in NEDP1 knockout cells and the modification of KR NEDD8 mutants, related to Figures 3, 4.

(A) U2OS cells transfected with pcDNA3 or the dominant negative cullin 5-Flag expression construct were seeded in 24 well plates and treated with etoposide as indicated for 15hrs before measurement of caspase 3/7 activity as before. Graph represents average values (*n*=2) +/- SEM.

(B) Parental or NEDP1 knockout U2OS cells (clones C6, H6) were treated with etoposide (50μM for 15hrs) and cell extracts were analysed with the indicated antibodies by western blotting.

(C) U2OS cells transfected as described in Methods with the indicated siRNAs, wild type and KR NEDD8 expression constructs, were used for nickel pull-downs. Eluates and total cell extracts were used for western blotting.

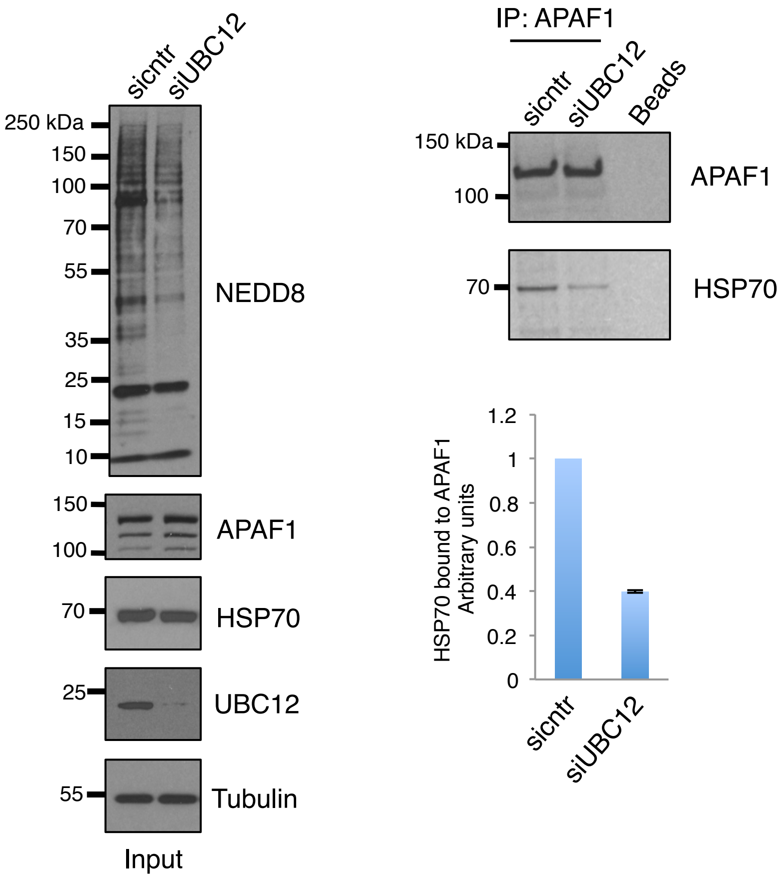

Figure S5. UBC12 knockout decreases the APAF1-HSP70 binding in H6 cells, related to Figure 5.
NEDP1 knockout U2OS cells (H6) were transfected either with control or UBC12 siRNAs. Extracts were used for APAF1 immunoprecipitations (IP) and western blotting as described in Fig. 5D. Extracts from the sicntr cells were used for the beads only IP. The HSP70 signal in the IPs was quantified and the average values (*n*=2) +/- SEM relative to the control is presented in the bottom right graph.

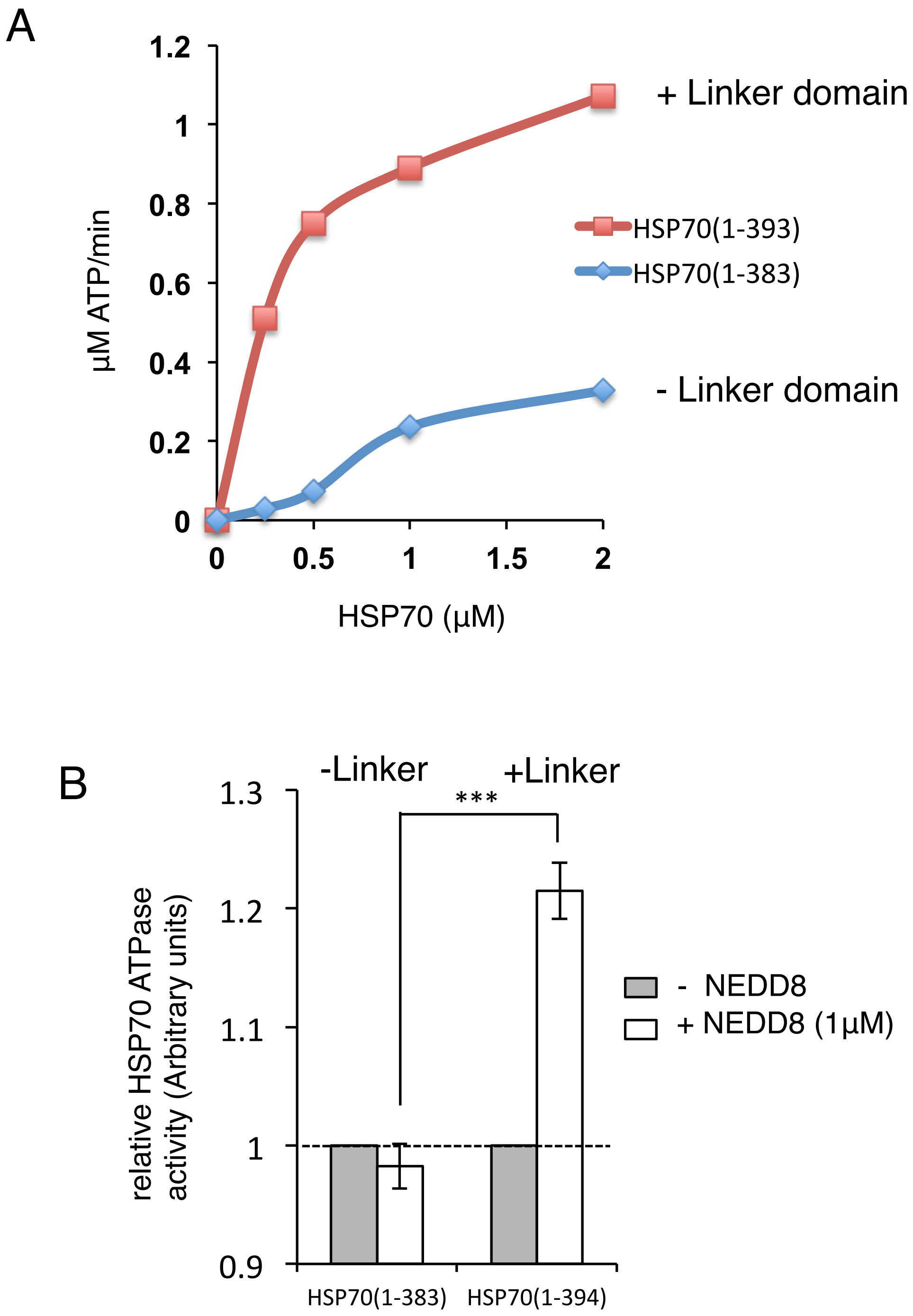

Figure S6. The Linker domain is critical for the stimulation of the HSP70 ATPase activity by NEDD8, related to Figure 6.

(A) The ATP hydrolysis activity of the HSP70 ATPase domain in the presence or absence of the Linker domain (383-393) was measured.

(B) The effect of NEDD8 on the HSP70 ATP hydrolysis activity in the presence or absence of the linker domain in HSP70. Average values (*n*=5) +/- SEM with p value <0.001.

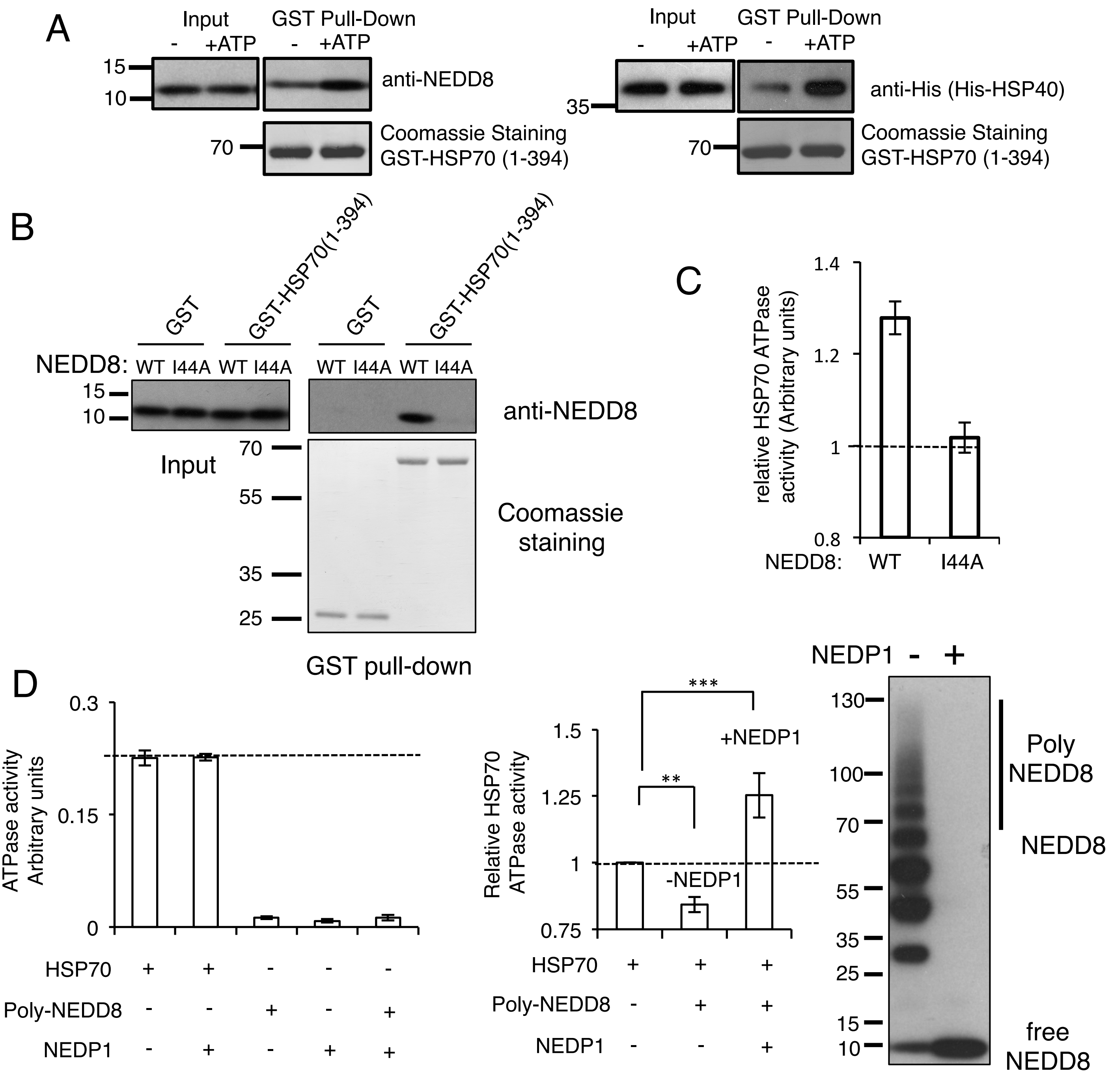

Figure S7. Characterisation of the effect of NEDD8 on HSP70 ATPase activity, related to Figure 6.

(A) ATP stimulates the HSP70 interaction with NEDD8 (left panel) or HSP40 (right panel) in GST-HSP70 pull-downs.

(B) The NEDD8 point mutant I44A fails to bind the HSP70 ATPase domain *in vitro* in a GST pull-down assay.

(C) The I44A mutation inhibits the NEDD8 stimulatory effect on HSP70 ATPase activity *in vitro*. Average values (n=5) +/- SEM with p value <0.001.

(D) Poly-NEDD8 conjugates synthesized *in vitro* using recombinant His_6-NEDD8 were isolated by Ni^{2+} pull-down and subjected to NEDP1 digestion (right panel) and processed as in Fig.6F. (n=5 +/- SEM, p values $** \leq 0.01$, $*** \leq 0.001$).
