# Supplementary material for: The Balance between Mono- and NEDD8-Chains Controlled by NEDP1 upon DNA Damage Is a Regulatory Module of the HSP70 ATPase Activity
Source: Cell Rep. 2019 Oct 1;29(1):212–224.e8. doi: 10.1016/j.celrep.2019.08.070 (PMC6899524; doi:10.1016/j.celrep.2019.08.070)
Supplement: Document S2. Article plus Supplemental Information [file mmc3.pdf]

Cell Reports

Article

The Balance between Mono- and NEDD8-Chains Controlled by NEDP1 upon DNA Damage Is a Regulatory Module of the HSP70 ATPase Activity

Graphical Abstract

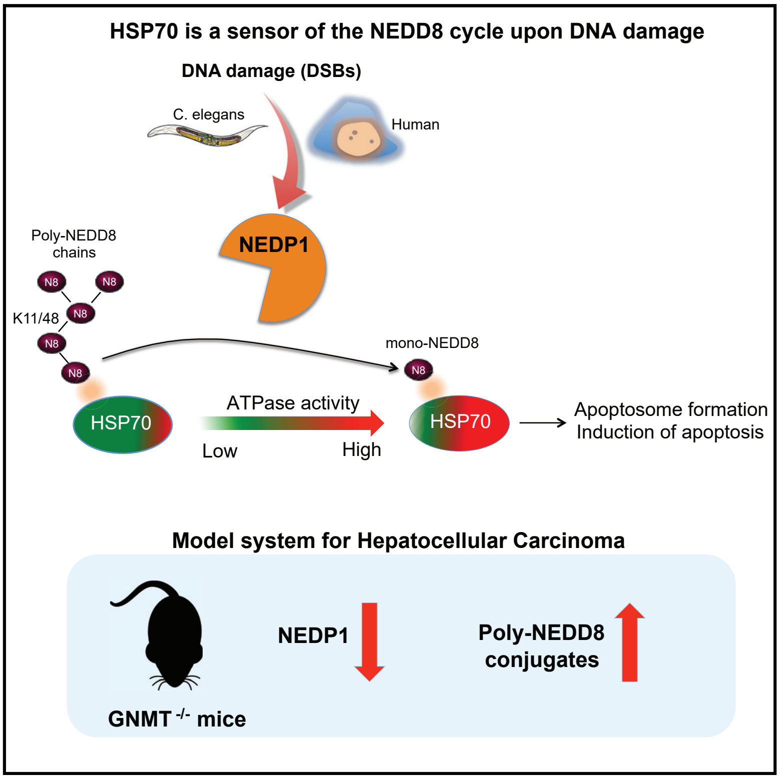

Authors

Aymeric P. Bailly, Aurelien Perrin, Marina Serrano-Macia, ..., M.L. Martinez-Chantar, Anton Gartner, Dimitris P. Xirodimas

Correspondence

aymeric.bailly@crbm.cnrs.fr (A.P.B.), dimitris.xirodimas@crbm.cnrs.fr (D.P.X.)

In Brief

The formation of ubiquitin and/or ubiquitin-like polymers is dynamically balanced by conjugating and de-conjugating enzymes. Bailly et al. report that the HSP70 chaperone is a sensor of the NEDD8 cycle. Conversion of NEDD8 chains into mono-NEDD8 by the de-NEDDylating enzyme NEDP1 upon DNA damage activates HSP70, which allows the formation of the apoptosome and apoptosis induction.

Highlights

- Restriction of NEDD8 chains by NEDP1 is required for DNA damage-induced apoptosis
- The HSP70 chaperone is a sensor of the balance between mono- and NEDD8 chains
- Mono-NEDD8 stimulates HSP70 activity, which allows the formation of the apoptosome
- NEDP1 levels are downregulated in mouse hepatocellular carcinoma

Bailly et al., 2019, Cell Reports *29*, 212–224
October 1, 2019 © 2019 The Author(s).
https://doi.org/10.1016/j.celrep.2019.08.070

CellPress

OPEN ACCESS
CellPress

Cell Reports
Article

The Balance between Mono- and NEDD8-Chains Controlled by NEDP1 upon DNA Damage Is a Regulatory Module of the HSP70 ATPase Activity

Aymeric P. Bailly,[1,*] Aurelien Perrin,[1] Marina Serrano-Macia,[2] Chantal Maghames,[1,4] Orsolya Leidecker,[1,4,5] Helene Trauchessec,[1] M.L. Martinez-Chantar,[2] Anton Gartner,[3] and Dimitris P. Xirodimas[1,6,*]
[1]CRBM, CNRS, Univ. Montpellier, UMR5237, Montpellier 34090, Cedex 5, France
[2]Liver Disease Laboratory, CIC bioGUNE, Centro de Investigación Biomédica en Red de Enfermedades Hepáticas y Digestivas (CIBERehd), 48160 Derio, Bizkaia, Spain
[3]Centre for Gene Regulation and Expression, College of Life Sciences, University of Dundee, Dow Street, Dundee DD1 5EH, UK
[4]These authors contributed equally
[5]Present address: Max Planck Institute for Biology of Ageing Joseph-Stelzmann-Str. 9b, 50931 Cologne, Germany
[6]Lead Contact
*Correspondence: aymeric.bailly@crbm.cnrs.fr (A.P.B.), dimitris.xirodimas@crbm.cnrs.fr (D.P.X.)
https://doi.org/10.1016/j.celrep.2019.08.070

SUMMARY

Ubiquitin and ubiquitin-like chains are finely balanced by conjugating and de-conjugating enzymes. Alterations in this balance trigger the response to stress conditions and are often observed in pathologies. How such changes are detected is not well understood. We identify the HSP70 chaperone as a sensor of changes in the balance between mono- and poly-NEDDylation. Upon DNA damage, the induction of the de-NEDDylating enzyme NEDP1 restricts the formation of NEDD8 chains, mainly through lysines K11/K48. This promotes APAF1 oligomerization and apoptosis induction, a step that requires the HSP70 ATPase activity. HSP70 binds to NEDD8, and, *in vitro*, the conversion of NEDD8 chains into mono-NEDD8 stimulates HSP70 ATPase activity. This effect is independent of NEDD8 conjugation onto substrates. The study indicates that the NEDD8 cycle is a regulatory module of HSP70 function. These findings may be important in tumorigenesis, as we find decreased NEDP1 levels in hepatocellular carcinoma with concomitant accumulation of NEDD8 conjugates.

INTRODUCTION

A key characteristic of ubiquitin and ubiquitin-like (Ubl) molecules is their ability to modify substrates as single moieties or in the form of polymeric chains. The extent and topology of polymeric chains is finely balanced by the coordinated action of conjugating and de-conjugating enzymes (Williamson et al., 2013). The activity of these enzymes is altered as part of the cellular response to stress and is de-regulated in pathological conditions including cancer, immunological diseases, and neurodegenerative diseases (Popovic et al., 2014). Hence, these enzymes are regarded as major targets for therapeutic intervention. The outcome of such de-regulation is the change in the equilibrium between the mono- and polymeric states of ubiquitin and Ubl modification. However, the pathways that sense such alterations are not well understood (Popovic et al., 2014; Williamson et al., 2013).

The Ubl molecule NEDD8 is highly conserved and essential in almost all tested organisms. Its functions have been characterized mainly through mono-NEDDylation of the cullin family of proteins and stimulation of the activity of cullin-RING ligases (CRLs), but also through modification of non-cullin substrates (Abidi and Xirodimas, 2015; Enchev et al., 2015). Defects in the NEDD8 cycle resulting in increased levels of NEDDylation have been reported in several types of cancers, including lung adenocarcinomas, squamous-cell carcinoma, and hepatocellular carcinoma (HCC), and inhibitors of the NEDD8 pathway are in phase II clinical trials (Abidi and Xirodimas, 2015; Barbier-Torres et al., 2015; Delgado et al., 2018). Protein NEDDylation is a reversible process. The NEDP1 (DEN1, SENP8) protease specifically processes NEDD8 into the mature form, required for the activation of NEDD8 by the NEDD8 E1 enzyme and additionally catalyzes de-NEDDylation of substrates (Abidi and Xirodimas, 2015; Enchev et al., 2015). While proteomic studies have indicated the formation of NEDD8 chains in cells, their regulation and biological function(s) are not well defined (Abidi and Xirodimas, 2015; Enchev et al., 2015). By combining studies in *C. elegans* and in human cells, we found a conserved role of the NEDD8 cycle in the DNA damage-induced apoptosis. The induction of NEDP1 upon DNA damage restricts the formation of NEDD8 chains mainly through lysines K11/K48 in the cytoplasm. This promotes the oligomerization of the apoptotic protease activating factor 1 (APAF1) and apoptosis induction. We found that de-NEDDylation is required for the release of the heat shock protein 70 (HSP70) chaperone from APAF1, a required step toward APAF1 oligomerization. HSP70 binds to NEDD8 and we mapped the ATPase domain as the binding site for NEDD8 on HSP70. Biochemical analysis shows that the balance between mono-NEDD8 and NEDD8 chains is a regulatory module for HSP70 function; mono-NEDD8 activates the ATPase activity of HSP70, which is counteracted upon NEDD8

This is an open access article under the CC BY license (http://creativecommons.org/licenses/by/4.0/).

polymerization. Restriction of poly-NEDDylation by NEDP1 restores the stimulatory effect of NEDD8 on HSP70 ATPase activity. The studies reveal that HSP70 is a sensor of changes in the NEDD8 cycle controlled by NEDP1. They also provide mechanistic insights on the role of poly-NEDDylation restriction as an activation signal for HSP70 function and apoptosis induction upon DNA damage. These findings may be relevant in pathology, as we found that NEDP1 protein levels are downregulated in a mouse model system for hepatocellular carcinoma with concomitant accumulation of NEDD8 conjugates. Collectively, the data provide a molecular basis for a potential suppressive role of NEDP1 in tumorigenesis through restriction of NEDD8 chains.

RESULTS

The De-NEDDylating Enzyme ULP-3/NEDP1 Restricts the Formation of K11/K48 NEDD8 Chains and Is Required for DNA Damage-Induced Apoptosis in *C. Elegans*

We identified *ulp-3* (Ubl protease-3, sequence Y48A5A.2, GenBank: NP_001023477.1) as the *C. elegans* homologous gene of human NEDP1 by reciprocal BLAST analysis (Figure 1A). ULP-3 has the catalytic triad His/Asp/Cys that defines the cysteine protease super-family (Figure 1A). *In vitro*, ULP-3 processes the NEDD8 C-terminal similarly to NEDP1, whereas *in vivo*, overexpression of ULP-3 in human cancer cells decreases NEDDylation of L11, a previously characterized NEDD8 substrate (Xirodimas et al., 2008) and has no effect on total ubiquitination. Both activities depend of the predicted catalytic C167 (Figures S1A and S1B). These results indicate that *C. elegans* ULP-3 is a bona fide NEDD8-specific protease and the *C. elegans* homologous protein to human NEDP1.

We obtained a deletion allele (*ulp-3* (*tm1287*)), within the reading frame, which abolishes ul*p-3* expression, shown by RNA and protein-level analysis (Figures S1C, S1D, and S1E). The *ulp-3* knockout (KO) animals are viable, and further systematic phenotypic characterization shows no defects in cell cycle progression, growth, and fertility compared to wild-type animals (data not shown). However, worms deleted or silenced for *ulp-3,* in contrast to wild-type animals, are almost completely resistant to the induction of apoptosis upon ionizing radiation (IR) in germ cells (Figures 1B and 1C). Therefore, ULP-3 is not essential for viability and development in *C. elegans* but it is required for the IR-induced apoptosis.

The apoptotic core pathway in *C. elegans* is responsible for both germ cell homeostasis and developmental programmed cell death (Bailly and Gartner, 2013; Lettre and Hengartner, 2006). We exploited the worm mutant *ced-1* (*e1735*), in which apoptotic corpse engulfment is severely impaired, allowing quantitation of developmental apoptosis. No difference in the number of apoptotic cells that persist in the head of L1 larvae between the *ced-1* single mutant and the *ulp-3; ced-1* double mutant was observed, suggesting a specific role for ULP-3 in germ cells apoptosis upon IR (Figure S2A). By using the ts *glp-4* mutant in which the germline is eliminated at the restrictive temperature or by analyzing dissected germlines, we found by qPCR and western blot analysis that *ulp-3* is preferentially expressed in germ cells, providing an explanation for the specific role of ULP-3 in the IR-induced apoptosis in germ cells (Figure S2B).

In response to double-strand breaks (DSBs), the main cellular circuitry involved in apoptosis induction is the DNA-damage checkpoint signaling pathway that ultimately triggers the highly conserved transcription activator p53, called CEP-1 in *C. elegans* (C. elegans p53-like 1) (Bailly and Gartner, 2013; Lettre and Hengartner, 2006). We analyzed multiple phenotypes indicative of an efficient activation of the DNA damage checkpoint signaling pathway: we determined the sensitivity of the *ulp-3* mutant to IR in a clonogenic survival assay indicative of defects in DSBs repair, assessed the cell cycle arrest induced by DNA damage, and quantitatively measured the induction of the two CEP-1 target genes, *egl-1* and *ced-13*, as readout of CEP-1/p53 activation. *ulp-3* deletion has no effect on DNA repair, cell cycle arrest, or CEP-1 activation upon IR (Figures S2C and S2D), suggesting that the cascade of events that follow the activation of the DNA damage checkpoint signaling pathway is not under ULP-3 regulation. We concluded that the branching point where ULP-3 intersects the DNA damage checkpoint signaling pathway is downstream or parallel to the *C. elegans* p53-like protein CEP-1.

Downstream of CEP-1 activation, the oligomerization of CED-4 (APAF1 homolog) into the apoptosome homomeric complex is the critical step for caspase 3-like protein (CED-3) activation and apoptosis induction (Qi et al., 2010). We monitored the effect of *ulp-3* deletion on CED-4 localization using a *C. elegans* strain stably expressing CED-4::GFP under its endogenous promoter and 3′UTR. CED-4::GFP displays perinuclear localization consistent with the pattern of endogenous CED-4 (Figure 1D). By using fluorescence recovery after photobleaching (FRAP) in living worms, we determined the mobile fraction of CED-4::GFP in the perinuclear area, as 19.24% ± 0.69%, which is compatible with the mobile fraction of a membranous or membrane-interacting protein (Wolter et al., 1997). Deletion of *ulp-3* significantly reduces the CED-4::GFP mobile fraction by nearly 40% (12.18% ± 1.03%) (Figures 1D and 1E). DNA damage by IR leads to the appearance of CED-4::GFP as punctate structures in the vicinity of the nuclear membrane, and this phenomenon was exacerbated in the *ulp-3* mutant (Figure 1F). FRAP analysis showed that these CED-4::GFP structures at the perinuclear membrane become completely immobile (data not shown). The observed defects in the CED-4 mobile fraction and apoptosis induction upon IR in the *ulp-3* mutant were restored to a nearly wild-type level upon transient *ned-8* RNAi treatment (Figures 1E and 1G). The data indicate that de-NEDDylation by ULP-3 increases the mobile fraction of CED-4 required for the activation of the apoptotic response to IR.

To gain mechanistic insights into the role of ULP-3 in the IR-induced apoptosis, we devised an unbiased proteomics approach to discover potential NEDD8 targets for ULP-3 upon DNA damage. Identification of diglycine (diGly) remnants left on lysine residues upon trypsin digestion of proteins by mass spectrometry demonstrates their modification with ubiquitin, NEDD8, or ISG15 (Kessler, 2013; Ordureau et al., 2015). We hypothesized that changes in the diGly signature upon deletion of the de-NEDDylating enzyme ULP-3 should specifically indicate

OPEN ACCESS
CellPress

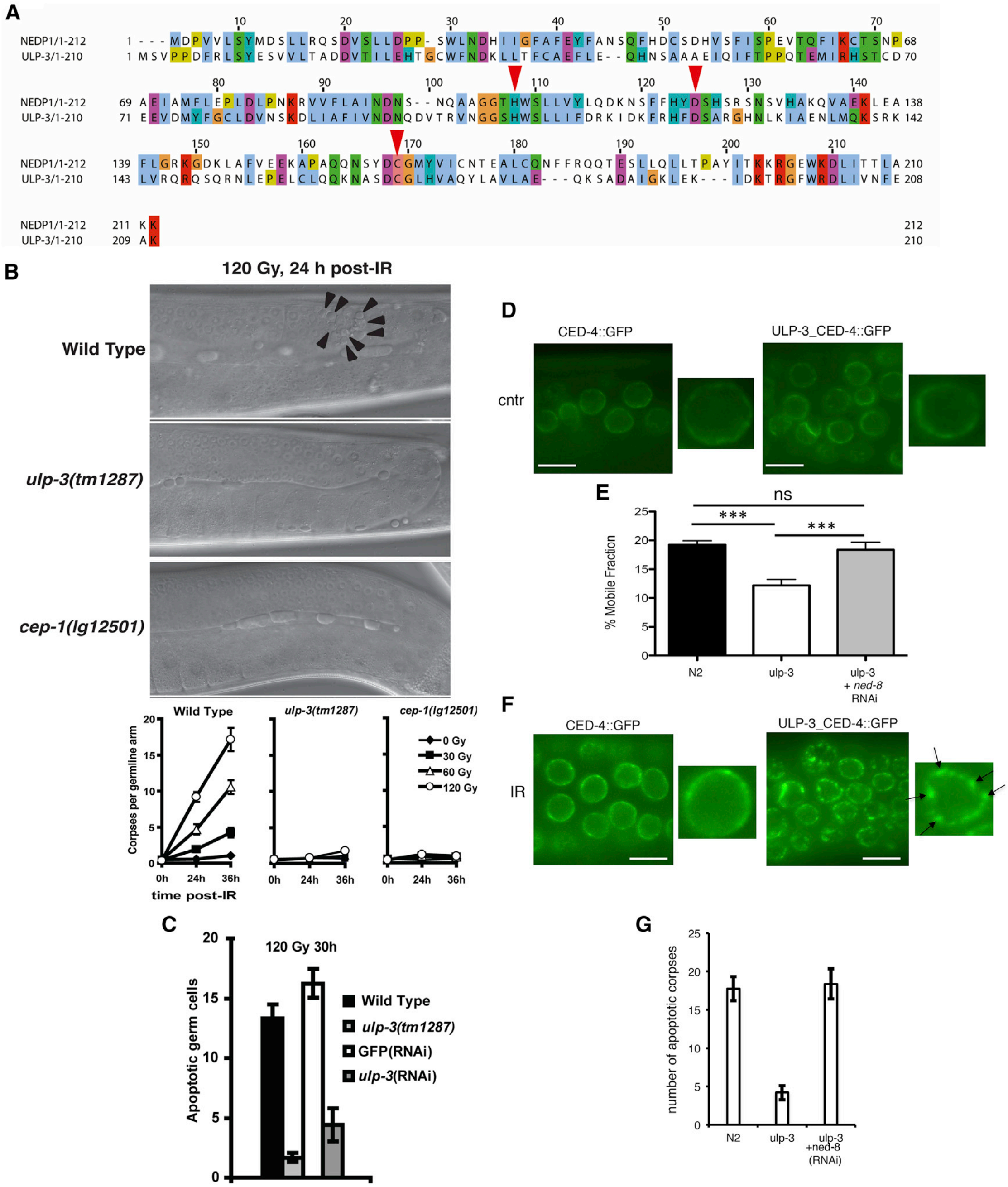

Figure 1. The De-NEDDylating Enzyme ULP-3 Is Required for DNA Damage-Induced Apoptosis in *C. elegans*

(A) ClustalWS alignment of human NEDP1 and *C. elegans* ULP-3 shows the conservation of the catalytic triad His106/Asp123/Cys167 (red arrows).

(B) Wild-type-, *ulp-3*-, and *cep-1*-deleted animals were irradiated as indicated. Germ cells were monitored through differential interference contrast (DIC) optics and apoptotic cells were counted. The average number of apoptotic cells for at least 15 animals of each genotype is plotted with error bars including SEM (bottom panel).

(C) Similar experiment as in (B), including RNAi treatments in wild-type animals as indicated.

(D) CED-4::GFP localization in wild-type and *ulp-3* mutant germ cells. Living worms were dissected and gonads immediately prepared for confocal microscopy.

(E) CED-4::GFP mobile fraction is determined by fluorescence recovery after photobleaching (FRAP) in the indicated genetic backgrounds and RNAi treatment. Average values (n = 20) of mobile fraction ± SEM (t test, $p \leq 0.001$).

(F) CED-4::GFP localization in the indicated backgrounds 24 h after 120 Gy of IR. Arrows indicate the CED-4::GFP punctate structures at the perinuclear area in ul*p-3* mutant germ cells.

(G) *ned-8* RNAi in *ulp-3*-deleted animals. Average number of apoptotic cells was determined as in (B) (n > 15) ± SEM. Scale bar, 3 μm.

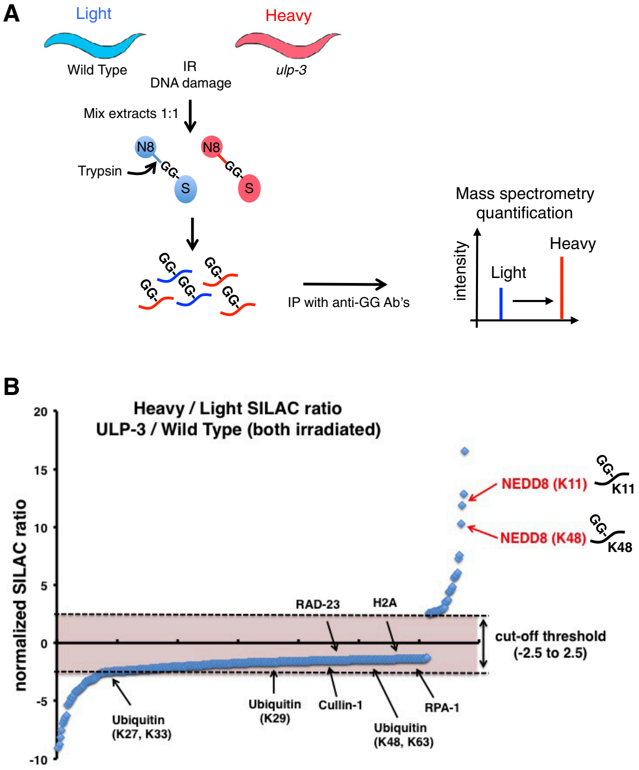

Figure 2. ULP-3 Restricts the Formation of K11/K48 NEDD8 Chains

(A) Proteomic SILAC strategy in *C. elegans* to identify ULP-3 targets upon DNA damage. Worms labeled with light (wild type) or heavy (*ulp-3* mutant) isotopic amino acids were exposed to 120 Gy of IR. Protein extracts were a mixed 1:1 ratio and after trypsin digestion diGly containing peptides were immunoprecipitated and analyzed by MS.

(B) Plot of normalized SILAC ratio of unique diGly peptides (top panel).

changes in the NEDD8 modification repertoire. We combined stable Isotope labeling with amino acids in nematodes (SILAC) (Larance et al., 2011) with the use of antibodies that recognize the diGly remnant on modified peptides (Kessler, 2013; Ordureau et al., 2015; Xu et al., 2010). Wild-type and *ulp-3*-deleted worms were labeled with light and heavy isotopes, respectively (Figure 2A). To specifically address the effect of ULP-3 on the NEDD8 proteome upon DNA damage, both sets of worms were exposed to IR. Extracts were mixed in a 1:1 ratio before immunoprecipitation with the anti-diGly antibody followed by tandem mass spectrometry (MS/MS) (Figure 2A). A total of 951 non-redundant diGly peptides were quantified, among which the abundance of 32 was significantly increased (> 2.5-fold) upon *ulp-3* deletion after IR (Table S1). Interestingly, no significant changes in the modification of multiple proteins involved in the DNA-damage response were observed (Figure 2B), consistent with the genetic characterization, suggesting that ULP-3 does not control the DNA damage checkpoint activation. By contrast, we observed a significant increase for the modification of NEDD8 on lysines 11 and 48 (K11/K48, SILAC ratios of 10.6 and 11.2, respectively) (Figure 2B), indicating that deletion of ULP-3 increases NEDD8 chain formation. We did not detect the modification of components of the NEDD8 machinery including NAE and UBC12, which was previously reported in NEDP1 knockout conditions in human cells and plants (Coleman et al., 2017; Keuss et al., 2019; Mergner et al., 2017). This may be a *C. elegans*-specific effect or due to the fact that IR was included in our proteomics experiments. The data highlight that a key function of ULP-3 upon DNA damage is to restrict the formation of NEDD8 chains through lysines K11/K48 (Figure 2B).

NEDP1 Is a DNA Damage-Induced Gene with a Conserved Role in DNA Damage-Induced Apoptosis in Human Cells

To determine and biochemically explore the conservation of our findings in *C. elegans*, we investigated in human cells the role of the ULP-3 homolog NEDP1 in the DNA damage-induced apoptosis. Knockdown of NEDP1 by small interfering RNA (siRNA) increased overall protein NEDDylation (Figure 3A). Importantly, the observed increase in NEDDylation depends on the canonical NEDD8-activating enzyme (NAE) and not on NEDD8 activation by the ubiquitin E1 enzyme Ube1, which occurs under proteotoxic stress (Hjerpe et al., 2012; Leidecker et al., 2012; Maghames et al., 2018) (Figure 3A, middle panel). A dramatic increase in protein NEDDylation with no effect on ubiquitination is observed in two different clones of U2OS (C6 and H6), where NEDP1 is deleted using CRISPR/Cas9 (Figures 3B and S3). Consistent with previous studies, NEDDylation of cullins was not dramatically affected, with the exception of cullin 5, for which NEDDylation was reduced in NEDP1 knockout cells (Figure 3B). By monitoring caspase 3/7 activation or Annexin V staining, we found that NEDP1 knockdown significantly decreases the IR-induced apoptosis (Figures 3C and 3D). Similar results were obtained using the NEDP1 knockout U2OS cells exposed to the chemical inducer of DSBs, etoposide (Figure 3E). Expression of a dominant negative construct for cullin 5 caused a small increase in the sensitivity to DSB-induced apoptosis, suggesting that the resistance of NEDP1 knockout cells to DSB-induced apoptosis is not due to defects in CRL5 function, as a consequence of the observed decrease in cullin 5 NEDDylation in NEDP1 knockout cells (Figure S4A). In addition, NEDP1 deletion does not affect either γH2AX or p53 stabilization upon DSBs (Figure S4B). Importantly, DSBs induce a late (24 h post IR) expression of the NEDP1 mRNA and protein levels, showing that NEDP1 is a DNA damage-responsive gene (Figure 3F). Consistent with the notion that NEDP1 does not control early events in the DNA damage response at the chromatin level, subcellular fractionation shows that both NEDP1 and the accumulated NEDD8 conjugates upon NEDP1 deletion are almost exclusively localized in the cytoplasm (Figure 4A). These findings are consistent with the observations in *C. elegans* showing that the upstream activation of the DNA damage checkpoint is unaffected by ULP-3 deletion. Based on the proteomic analysis in *C. elegans*, we tested in human cells the role of K11 and K48 in the induction of apoptosis upon DSBs. We decided to test the double K11/K48R mutant, as single KR NEDD8 mutants do not show a significant impact on NEDDylation in NEDP1 knockdown cells, consistent with previous studies (Sui et al., 2015). The K27R NEDD8 mutant is deficient in NEDDylation as it is reported to indirectly block NEDD8

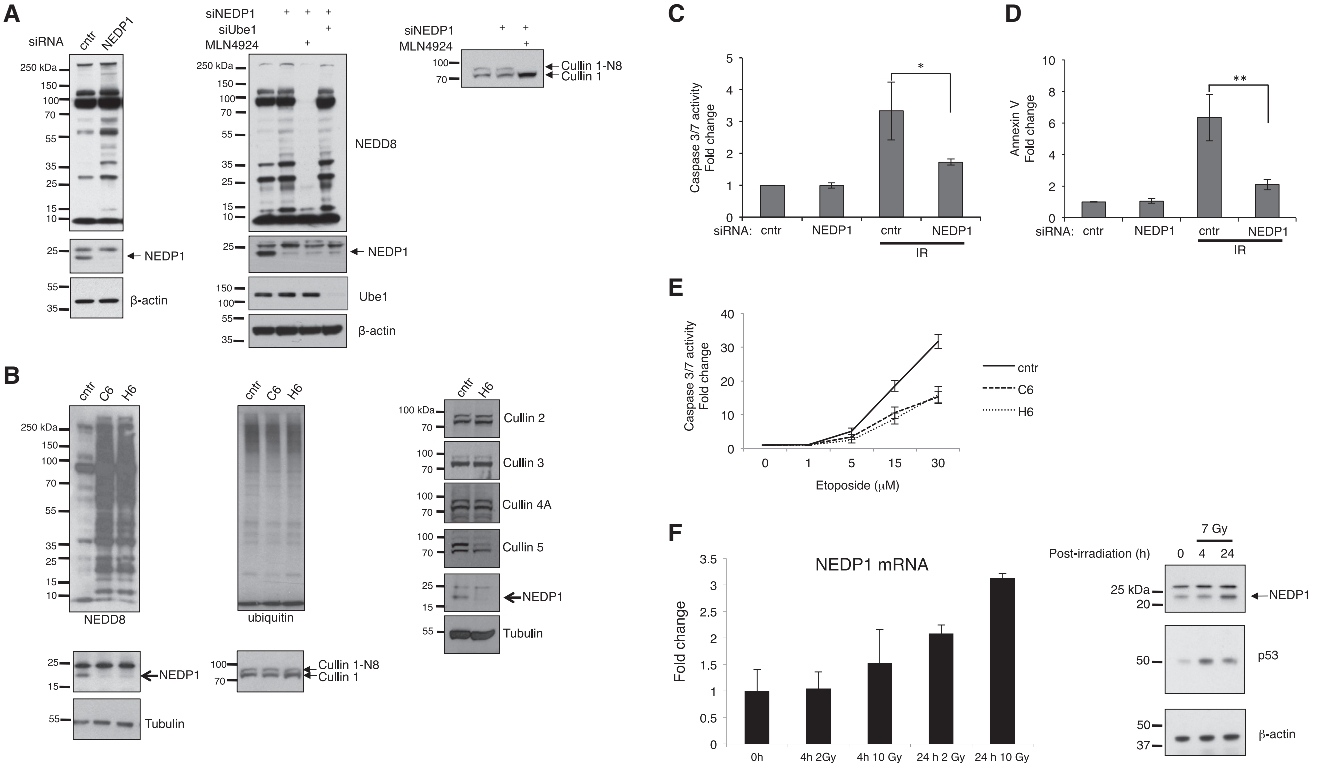

Figure 3. Conserved Role for NEDP1 in the DNA Damage-Induced Apoptosis

(A) Western blotting of extracts from U2OS cells transfected with siRNAs and treated with the NEDD8 E1 inhibitor MLN4924 (1 μM, 15 h) as indicated. Extracts were re-analyzed with cullin-1 antibodies (right panel).

(B) Western blot analysis of extracts from parental U2OS or U2OS NEDP1 knockout clones (C6 and H6).

(C and D) Similar experiment as in (A), except cells were treated with IR (7 Gy) and analyzed either for caspase 3/7 activity (C) or AnnexinV staining (D) 9 h later.

(E) Caspase 3/7 activity in parental or NEDP1 knockout U2OS cells exposed to etoposide for 15 h. Average values (n = 3) ± SEM.

(F) MCF7 cells were irradiated with 2 or 10 Gy for the indicated times. Quantitative real-time PCR for NEDP1 was carried out as described in the STAR Methods. The experiments were performed in triplicates; average values ± SEM (left panel). Western blot analysis in extracts of MCF7 cells treated as indicated (right panel).

conjugation due to the covalent modification of lysine residues in the NEDD8 E2-conjugating enzyme UBC12 (Figure S4C) (Sui et al., 2015).

We generated U2OS cells stably expressing either a wild-type or K11/K48R His_6-NEDD8 mutant at endogenous levels (Liu and Xirodimas, 2010) (Figure 4B). As expected in the presence of wild-type NEDD8, knockdown of NEDP1 increases NEDDylation and prevents the induction of apoptosis upon DSBs (Figures 4B and 4C). However, the K11/K48R NEDD8 mutant impaired the increase in NEDDylation upon NEDP1 knockdown and partially restored (up to 70%) the IR-induced apoptosis (Figures 4B and 4C). In combination with the proteomic analysis in *C. elegans*, the data suggest that the de-NEDDylation of NEDD8 chains via their K11/K48 linkage is the main and conserved function of ULP-3/NEDP1 that is required for the induction of apoptosis upon DNA damage.

NEDP1 Is Required for HSP70-Mediated APAF1 Oligomerization upon DNA Damage

Based on the findings in *C. elegans* on the role of ULP-3 on CED-4 mobility, we followed a biochemical approach in human cells to determine the role of NEDP1 on APAF1 activation upon DSBs. We monitored by sucrose gradient sedimentation the oligomerization of APAF1, indicative of the formation of the apoptosome (Bratton and Salvesen, 2010). While in control cells DSBs cause the formation of APAF1 oligomers, in NEDP1 knockout cells APAF1 oligomerization was severely impaired and APAF1 remained in its monomeric state (Figure 5A). The findings in *C. elegans* and human cells indicate that ULP-3/NEDP1 through de-NEDDylation promotes the formation of the active apoptosome upon DSBs.

APAF1 oligomerisation is a dynamic and key event in the initiation of apoptosis (Bratton and Salvesen, 2010; Cain et al., 1999; Rodriguez and Lazebnik, 1999). Among several control mechanisms, the binding of the HSP70 to APAF1 is an important regulatory event in APAF1 oligomerization. More specifically, the activation and release of HSP70 from APAF1 upon stress is required for the apoptosome formation and induction of apoptosis (Beere et al., 2000; Saleh et al., 2000). However, the signals, mechanism(s), and co-chaperone(s) involved in HSP70 activation under these conditions remain unclear.

Consistent with the previous studies (Beere et al., 2000; Saleh et al., 2000), we found that DSBs caused the decrease of HSP70 binding to APAF1 in control U2OS cells (Figure 5B). However, in

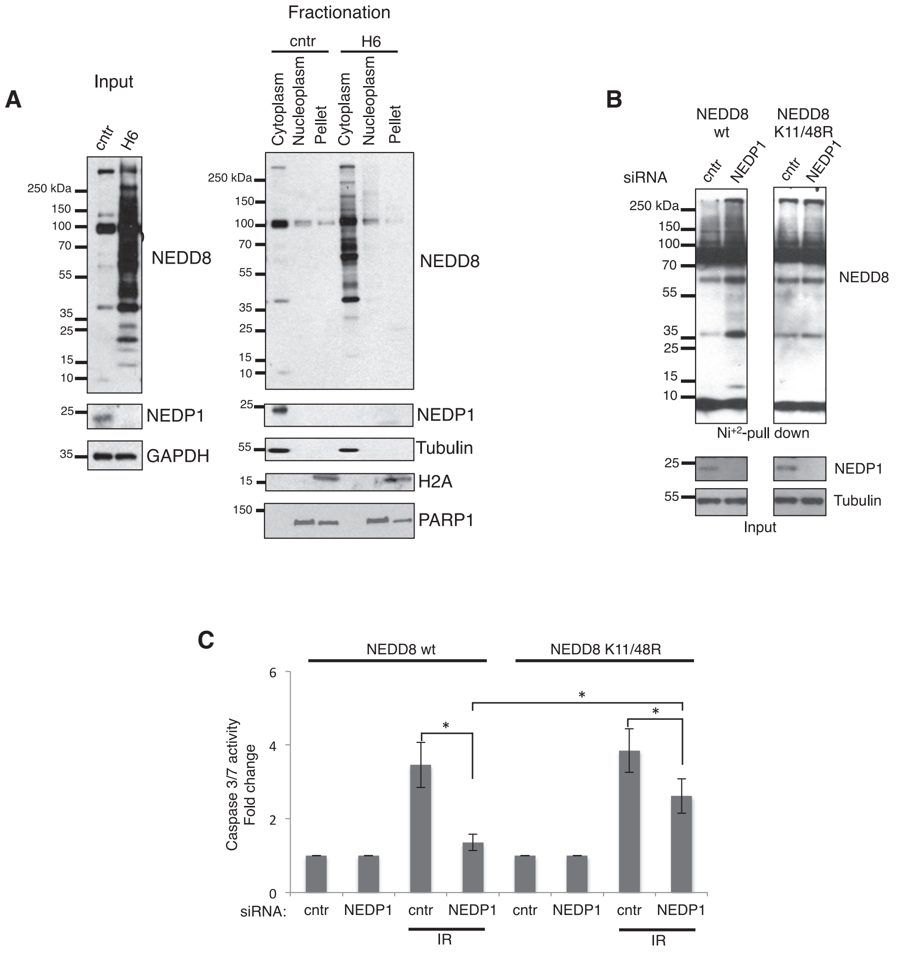

Figure 4. Restriction of K11/K48 NEDD8 Chains in the Cytoplasm Is Required for DNADamage-Induced Apoptosis

(A) Subcellular fractionation in parental or H6 NEDP1 knockout U2OS cells and western blotting for the indicated proteins.

(B) U2OS cells stably expressing either wild-type or K11/K48R His_6-NEDD8 mutant were transfected with the indicated siRNAs. Isolated His_6-NEDD8 conjugates were blotted for NEDD8. Expression levels of indicated proteins in total cell extracts (input).

(C) Similar experiment as in Figure 3C. Average values (n = 3) ± SEM; *p $\leq$ 0.05 and **p $\leq$ 0.01.

the NEDP1 knockout H6 cells an increased HSP70-APAF1 binding was observed both in unstressed and DSB stress conditions (Figure 5B). We investigated several possibilities of how the increase in NEDDylation observed in NEDP1 knockout cells impacts on the HSP70-APAF1 interaction, including assays monitoring the potential NEDDylation of HSP70 and APAF1. Under endogenous levels of NEDD8 expression and denaturing purification conditions that prevent non-covalent interactions, we did not detect the covalent modification of either HSP70 or APAF1 with NEDD8. In contrast, we found as the most consistent observation the interaction of HSP70 with the accumulated NEDD8 conjugates in H6 NEDP1 knockout cells (Figure 5C). This suggests that the binding of HSP70 with poly-NEDDylated conjugates in NEDP1 knockout cells compromises the release of HSP70 from APAF1. Consistent with this hypothesis, inhibition of poly-NEDDylation in NEDP1 knockout cells, either with the specific NEDD8 E1 inhibitor MLN4924 or with NEDD8 siRNA, reduced the binding of HSP70 to APAF1 (Figure 5D). Similar results were obtained with siRNAs against the NEDD8 E2-conjugating enzyme UBC12 (Figure S5). To specifically test the role of NEDD8 chain formation through K11/K48 in the HSP70-APAF1 interaction, we compared the binding of HSP70 to APAF1 in control or NEDP1 knockdown cells stably expressing either wild-type or the K11/K48R His_6-NEDD8 mutant. Consistent with the experiment in Figure 5B, NEDP1 knockdown in wild-type NEDD8-expressing cells, caused the accumulation of NEDD8 conjugates and the increase of the HSP70-APAF1 binding, compared to control cells. In contrast, in cells expressing the K11/K48R mutant the accumulation of NEDD8 conjugates upon NEDP1 knockdown was impaired and the HSP70-APAF1 binding was restored almost to control levels (Figure 5E). These data, in combination with the observed partial rescue of the apoptosis response in NEDP1 knockdown cells expressing K11/K48R (Figure 4C), suggest that the accumulation of NEDD8 chains through K11/K48 upon NEDP1 deletion or knockdown compromises the release of HSP70 from APAF1 and subsequently the apoptosis induction upon DSBs.

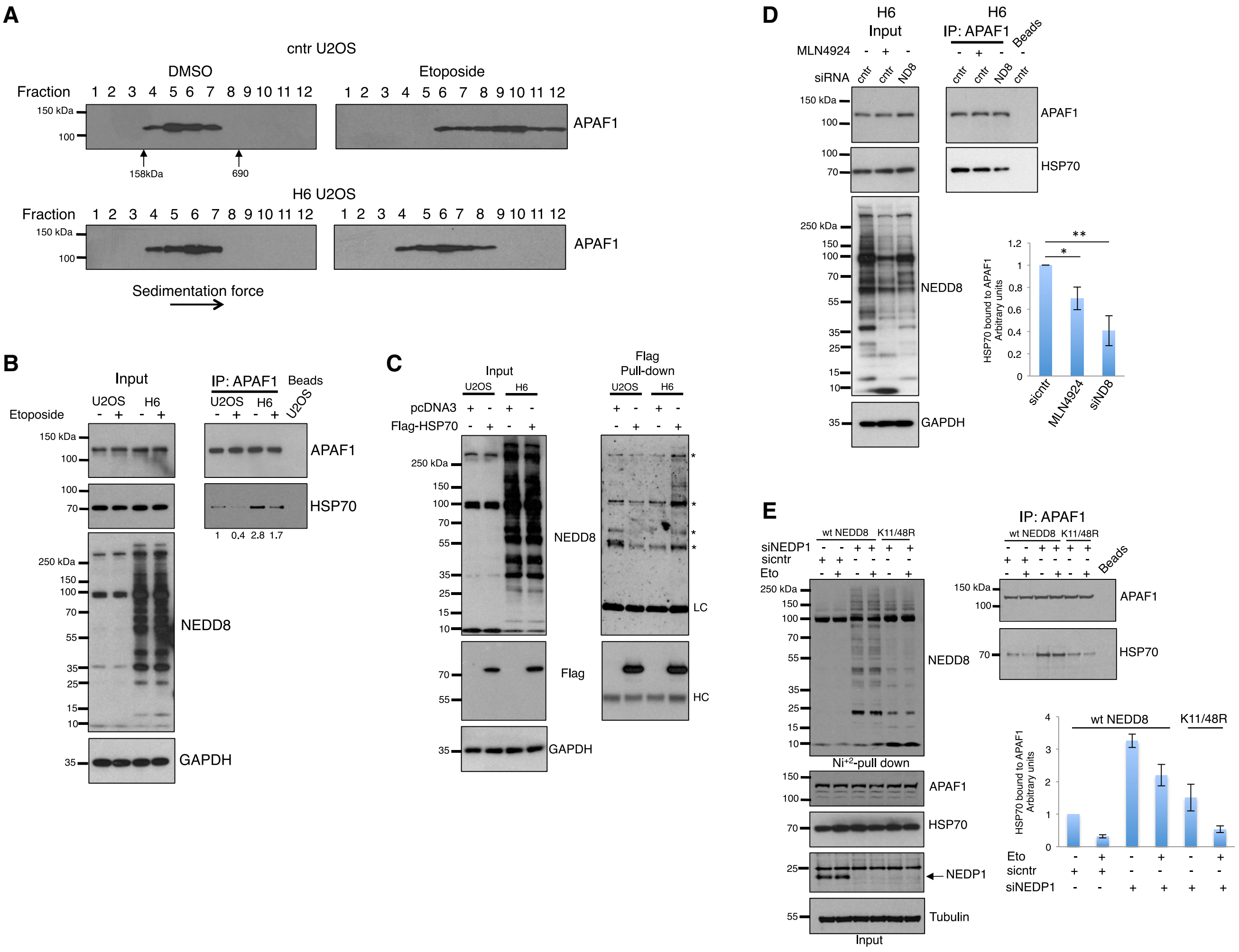

Figure 5. NEDP1 Is Required for HSP70-Mediated APAF1 Oligomerization upon DNA Damage

(A) Parental or H6 cells were either untreated (dimethyl sulfoxide [DMSO]) or exposed to etoposide (50 μM) for 15 h before extracts were subjected to sucrose gradient centrifugation. Fractions were analyzed by western blotting for APAF1. Arrows indicate the separation of Aldolase (158 kDa) and thyroglobulin (690 kDa).

(B) Parental or H6 cells were treated as in (A) and extracts were used for immunoprecipitation (IP) with APAF1 antibodies. Total cell extracts (input) and IPs were analyzed by western blotting with the indicated antibodies.

(C) Parental or H6 NEDP1 knockout U2OS cells stably expressing 3× FLAG-HSP70 were used for immunoprecipitations and western blotting as indicated. Asterisks indicate non-specific bands. LC and HC are shown by light and heavy chains, respectively.

(D) H6 cells were transfected with control (cntr) or NEDD8 (N8) siRNAs for 48 h. MLN4924 (1 μM) was added for 15 h. Signals for HSP70 were quantified with Image Gauge. Graph represents the average values (n = 4) ± SEM; *p ≤ 0.05, **p ≤ 0.01.

(E) U2OS cells stably expressing either wild-type or K11/K48R His$_6$-NEDD8 were transfected with siRNAs as described in the STAR Methods and were treated with etoposide (30 μM, 15 h) as indicated. Extracts were used for nickel pulldowns and IPs as before and western blotting was performed with the indicated antibodies. Graph represents the average values (n = 2) ± SEM.

NEDD8 Binds to the ATPase Domain of HSP70 and Stimulates HSP70 ATPase Activity

We then followed a biochemical approach to further characterize the NEDD8-HSP70 binding. Using recombinant proteins, we found a direct interaction between NEDD8 and HSP70 *in vitro* (Figures 6A and 6B). Mutational analysis showed that NEDD8 binds the ATPase domain of HSP70, between amino acids 190-394, which overlaps with the HSP40 J-domain binding region within the HSP70 ATPase domain (Figures 6B and 6C) (Ahmad et al., 2011). Furthermore, the HSP70 (190-394) fragment can also pull down NEDD8 conjugates from NEDP1 knockout cells (Figure 6D). While the *in vitro* analysis shows that HSP70 interacts with mono-NEDD8, the *in vivo* analysis suggests a preference of HSP70 for high-molecular-weight poly-NEDD8 conjugates.

ATP hydrolysis by HSP70 is a critical regulatory element for the interaction of HSP70 with client proteins, which is stimulated by HSP70 co-chaperones, including HSP40 (Kityk et al., 2012; Mayer and Bukau, 2005; Palleros et al., 1991; Zhuravleva et al., 2012). We found that mono-NEDD8 but not ubiquitin stimulates the ATPase activity of HSP70 to a similar extent as HSP40, indicating an important role for NEDD8 in HSP70

OPEN
ACCESS
CellPress

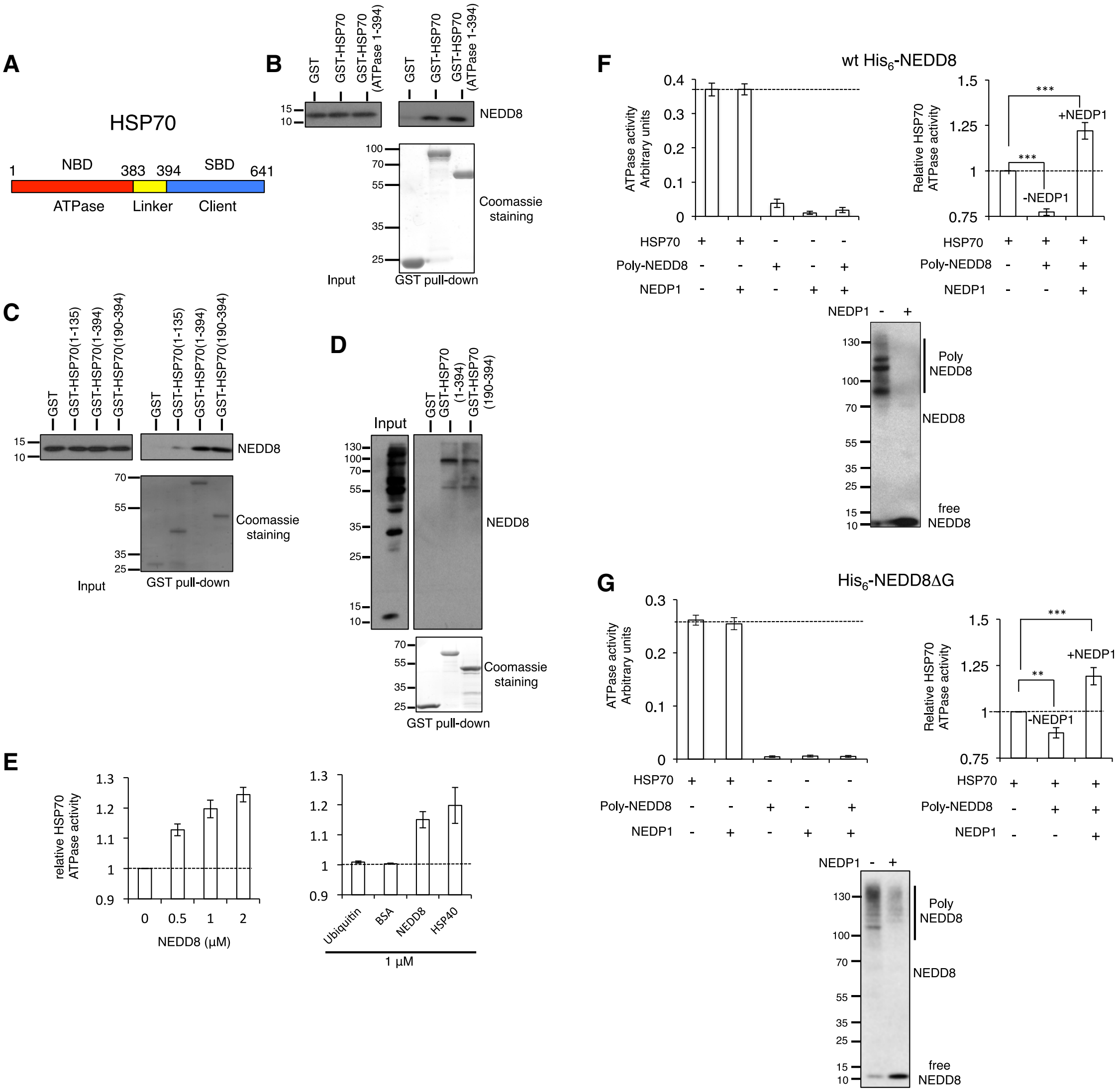

Figure 6. Mono-NEDD8 Stimulates the ATP Hydrolysis Activity of HSP70, Which Is Compromised upon Poly-NEDDylation

(A) Representation of the nucleotide binding domains (NBDs) and substrate binding domains (SBDs) in HSP70.

(B) Recombinant GST or various indicated GST-HSP70 constructs were incubated with recombinant NEDD8. Input and glutathione S-transferase (GST) pulldowns were analyzed by western blotting with anti-NEDD8 antibody.

(C) Experiment performed as in (B).

(D) Cells extracts from H6 NEDP1 knockout cells were incubated with GST, GST-HSP70 (1-394), or GST-HSP70 (190-394). Input and GST pulldowns were analyzed by western blotting with anti-NEDD8 antibody.

(E) The stimulation of the ATP hydrolysis activity of HSP70 was measured in the presence of increasing concentrations of NEDD8 (n = 3 ± SEM) (left). The relative stimulation of HSP70 ATPase activity by NEDD8 was compared to that of HSP40 (DNAJB4); ubiquitin and BSA were used as negative controls (n = 3 ± SEM) (right).

(F) Poly-NEDD8 conjugates were isolated from H6 NEDP1 knockout cells stably expressing His$_6$-NEDD8 by Ni^{2+} pulldown. Poly-NEDD8 conjugates were subjected to NEDP1 digestion (bottom panel) and tested in a HSP70 ATP hydrolysis activity assay (right panel, n = 5 ± SEM). Neither poly-NEDD8 conjugates nor recombinant NEDP1 carry contaminant ATP hydrolysis activity (left graph).

(G) Similar experiment as in (F) with the exception that H6 NEDP1 knockout U2OS cells were transfected with the His$_6$-NEDD8ΔG (n = 5 ± SEM); **$p \leq 0.01$, ***$p \leq 0.001$.

function regulation (Figures 6E and S6). ATP stimulated the NEDD8-HSP70 interaction, suggesting the preference of NEDD8 for the ATP-loaded state of HSP70 (Figure S7A), consistent with the stimulatory effect of NEDD8 on HSP70 ATPase activity. Mutation of a single amino acid (I44A) within the hydrophobic patch of NEDD8 prevented both the NEDD8-HSP70 interaction and the stimulatory effect of NEDD8 on HSP70 ATPase activity, showing the specific and direct effect of NEDD8 on HSP70 function under the purification conditions used (Figures S7B and S7C).

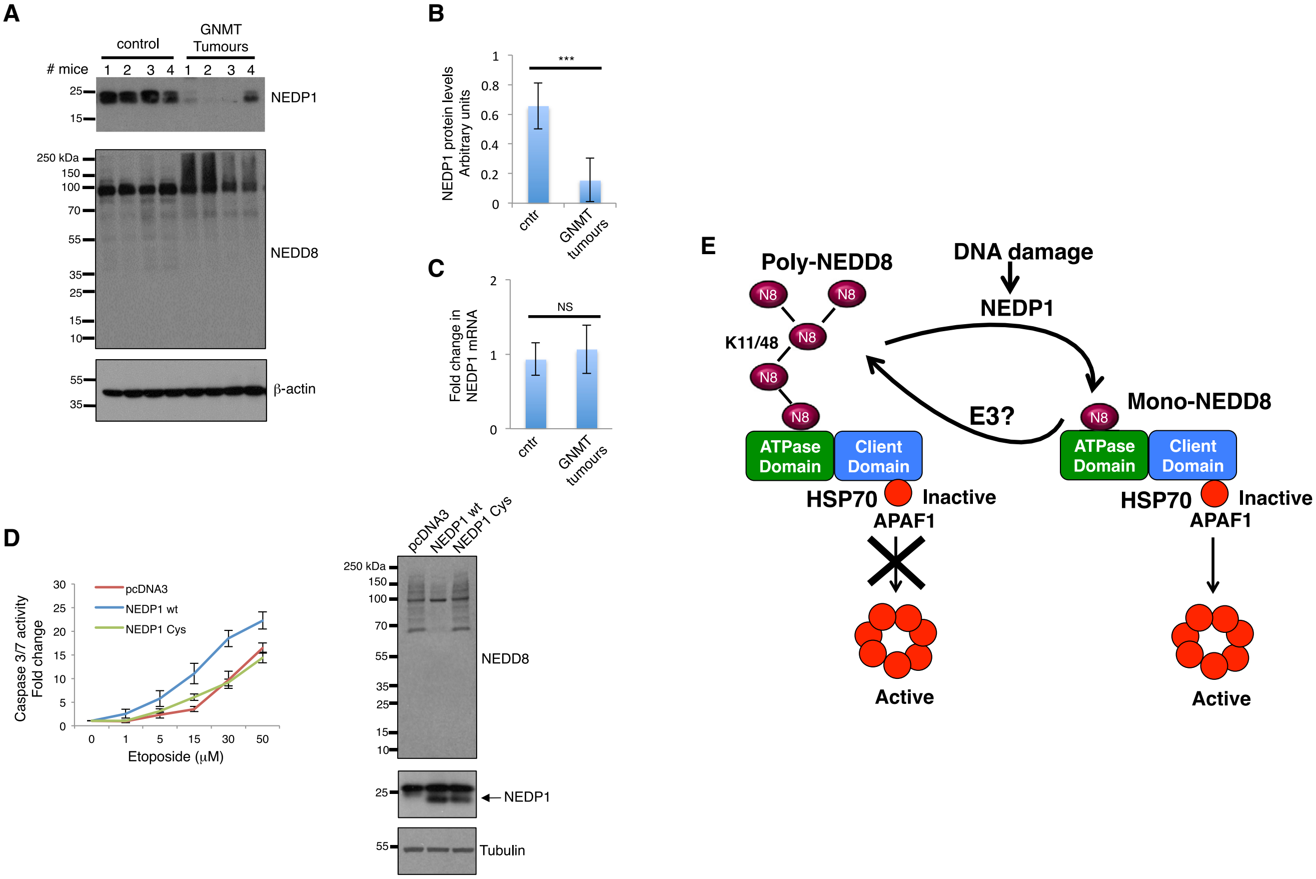

Figure 7. NEDP1 Levels Are Downregulated in Hepatocellular Carcinoma

(A) Liver tissue extracts from control or GNMT knockout tumoral group mice were analyzed for the levels of NEDP1 and NEDD8 by western blotting. B-actin was used as loading control.

(B) Quantitation of NEDP1 protein levels shown in (A). Data show the average of the NEDP1/β-actin ratio of four tested samples ± SEM; ***$p \leq 0.001$.

(C) Quantitative real-time PCR for nedp1 mRNA was carried out as described in the STAR Methods. Values represent the average (n = 8) ± SEM.

(D) Isolated GNMT tumor cells were transfected with wild-type or C163A inactive NEDP1 expression constructs. Western blot analysis of extracts from transfected cells (left panel). Transfected cells were seeded in 24-well plates, treated with etoposide as indicated, and caspace 3/7 activity was measured as before. Graph represents the average (n = 3) ± SEM (right panel).

(E) Model for the role of the NEDD8 cycle in HSP70 activity regulation and apoptosis induction upon DNA damage. Poly-NEDD8 chains (distinct K11, K48, or branched linkages) interact with HSP70 and prevent the release and oligomerization of APAF1 into an active complex. The induction of NEDP1 upon DNA damage restricts poly-NEDD8 chains into mono-NEDD8, which activates HSP70 ATPase activity resulting in APAF1 oligomerization and activation. The balance between mono- and poly-NEDDylation controlled by NEDP1 acts as a regulatory module for HSP70 function.

Poly-NEDDylation Blocks the Stimulatory Effect of Mono-NEDD8 on HSP70 ATPase Activity

We next determined the role of poly-NEDDylation in HSP70 ATPase function. We isolated poly-NEDD8 conjugates either from NEDP1 knockout cells stably expressing His_6-NEDD8 (Figure 6F) or from *in vitro* reactions using recombinant His_6-NEDD8 (Figure S7D). We employed high-salt purification conditions to ensure that no contaminant ATPase activity was co-purified (Figure 6F). In contrast to mono-NEDD8, the addition of poly-NEDD8 conjugates did not stimulate the HSP70 ATPase activity; rather a reproducible decrease was observed (Figures 6F and S7D). However, upon de-conjugation with the addition of recombinant NEDP1, an increase in HSP70 ATPase activity was observed (Figures 6F and S7D). We performed a similar experiment as in Figure 6F, transfecting instead NEDP1 knockout cells with a His_6-NEDD8 mutant deleted of its C-terminal glycine (His_6-NEDD8ΔG). This mutant is deficient in modifying substrates but itself can be modified by endogenous wild-type NEDD8, allowing the isolation of NEDD8 chains that are not attached to substrates (unanchored). Similarly to the data obtained with the wild-type NEDD8 chains, unanchored NEDD8 chains inhibited HSP70 ATPase activity, which was induced upon de-conjugation by NEDP1 (Figure 6G). This shows the direct role of NEDD8 chain processing by NEDP1 on HSP70 ATPase activity regulation, which is independent on the conjugation of NEDD8 to substrates.

NEDP1 Is Downregulated in Mouse HCC

The NEDD8 pathway is upregulated in several types of tumors, which often acquire resistance to the chemotherapy used

(Abidi and Xirodimas, 2015). Based on our observations, the accumulation of NEDD8 chains upon NEDP1 deletion compromises the induction of apoptosis upon DNA damage. However, the patho-physiological relevance for these findings remains unknown. We determined the expression of NEDP1 in the glycine N-methyltransferase (GNMT) knockout pre-clinical mouse model of HCC. GNMT is a tumor suppressor and deletion in mice results in the development of HCC (Martínez-Chantar et al., 2008). We chose this system as previous studies showed upregulation in protein NEDDylation in *GNMT*−/−-derived HCC tumors (Delgado et al., 2018). We found that NEDP1 protein levels in liver tissue extracts are dramatically reduced in all tested HCC animals compared to control animals (Figures 7A and 7B). The observed decrease is not due to changes in *nedp1* mRNA levels, suggesting a post-transcriptional mechanism (Figure 7C). Importantly, the decrease in NEDP1 levels is accompanied by a significant increase in NEDDylation, particularly for the high-molecular-weight conjugates, indicative of poly-NEDD8 conjugates (Figure 7A). To specifically test the effect of the reduced levels of NEDP1 in the response of HCC tumors to DSB-induced apoptosis, we expressed either wild-type human NEDP1 or the catalytic C163A mutant in cells isolated from HCC and expose them to etoposide. We found that expression of the wild-type but not of the catalytic inactive mutant reduces NEDDylation in these cells and increases their sensitivity to etoposide (Figure 7D). The data indicate that the decrease in NEDP1 levels induced during liver tumorigenesis compromises the sensitivity of these tumors to DSBs and provides a molecular mechanism for the reported increase of NEDDylation in these tumors.

DISCUSSION

The role of ubiquitin and SUMO modification in the DNA-damage response is established at the chromatin level, where upon recruitment they initiate complex signaling events for DNA repair and/or the induction of apoptosis (Jackson and Durocher, 2013; Zhao et al., 2014). Nuclear functions of NEDD8 required for DNA repair have also been reported through NEDDylation of cullins and non-cullin targets, including histones H2A, H4, and PCNA (Li et al., 2014; Ma et al., 2013; Guan et al., 2018). In this study, the combination of *C. elegans* genetics and biochemistry in human cells reveals a cytoplasmic role for NEDD8, as a protein quality control pathway of the apoptosome formation during the DNA damage-induced apoptosis.

In addition to the de-conjugating activity, NEDP1 also acts as a NEDD8-specific C-terminal hydrolase, exposing the di-glycine motif before NEDD8 activation by NAE (Abidi and Xirodimas, 2015; Enchev et al., 2015). The NEDD8 pathway is essential both in *C. elegans* and in humans, so the absence of lethality upon NEDP1 deletion indicates the presence of additional hydrolases that can process NEDD8. While NEDP1 is reported to control the mono-NEDDylation of both cullin and non-cullin substrates (Aoki et al., 2013; Broemer et al., 2010; Christmann et al., 2013; Coleman et al., 2017; Mergner et al., 2015; Watson et al., 2010; Xirodimas et al., 2008), the presented analysis shows that the restriction of NEDD8 chains in the cytoplasm is a key activity for ULP-3/NEDP1 that is required for the induction of apoptosis upon DNA damage. Previous studies identified multiple lysines as potential sites for NEDD8 chain formation including K11 and K48 (Coleman et al., 2017; Kim et al., 2011; Xirodimas et al., 2008). Mutational analysis indicates that the double mutation of K11 and K48 is required to significantly impair the accumulation of NEDD8 conjugates in NEDP1 knockdown cells. This suggests the formation of distinct but redundant NEDD8 polymers either through K11 or K48, or the formation of branched NEDD8 polymers through these lysines.

Initiation of the apoptotic process relies on the assembly of the apoptosome, which comprises several proteins including APAF1. Oligomerization of APAF1 provides the scaffold for the association and activation of caspases for apoptosis execution. Our studies suggest that NEDP1-dependent de-NEDDylation promotes, at least partially, the release of HSP70 from APAF1, which is regarded as an important step toward APAF1 oligomerization (Beere et al., 2000; Saleh et al., 2000). Association of HSP70 with its binding partners is mainly controlled by the N-terminal ATPase activity of HSP70. The intrinsic ATPase activity of HSP70 is low but it is stimulated by interacting co-chaperones, which increase the turnover of HSP70 binding to the substrate (Mayer and Bukau, 2005; Young, 2010). The biochemical analysis indicates that mono-NEDD8 has characteristics of an HSP70 co-chaperone as (1) NEDD8 interacts within the same region in the ATPase domain of HSP70 as the J domain of the HSP40 co-chaperone and the binding is stimulated by ATP (Figures 6C and S7A); (2) NEDD8 stimulates the ATPase activity of HSP70 to a similar extent as HSP40 (Figure 6E); and (3) the stimulatory effect of NEDD8 depends on the presence of the so-called linker domain in HSP70, a typical characteristic of co-chaperones (Jiang et al., 2007) (Figure S6). However, upon NEDD8 polymerization the stimulatory effect of NEDD8 on HSP70 ATPase activity is severely compromised, despite the interaction of HSP70 with poly-NEDD8 conjugates. Thus, the balance between mono-NEDD8 and NEDD8 chains is a regulatory element for HSP70 function. Future structural studies should provide detailed insights for the role of NEDD8 in HSP70 ATPase activity stimulation and how this effect is compromised upon NEDD8 polymerization.

The biochemical analysis proposes a mechanism for the role of the NEDD8 cycle on APAF1 oligomerization and DNA-damage-induced apoptosis; the induction of NEDP1 upon DNA damage restricts poly-NEDD8 conjugates into mono-NEDD8, creating the stimulatory signal for the HSP70 ATPase activity and apoptosome formation (Figure 7E). Consistent with this notion, accumulation of poly-NEDD8 conjugates upon the deletion of NEDP1 compromises the required release of HSP70 from APAF1 and subsequently APAF1 oligomerization, which are direct biological outcomes of defective HSP70 function. An interesting arising model is the role of HSP70 as sensor of the NEDD8 cycle. The extent and possibly the topology of poly-NEDDylation controlled by NEDP1 may indeed act as a ‘‘rheostat’’ to finely tune HSP70 chaperone activity (Figure 7E). The studies support a paradigm where NEDP1 acts as the molecular link to functionally connect the NEDD8 cycle with the HSP70 chaperone machinery upon DNA damage.

While we cannot exclude that the NEDDylation of a yet unidentified target(s) is part of the presented signaling cascade,

the *in vitro* data show that de-polymerization of NEDD8 chains is sufficient to activate HSP70 independently of a NEDD8 substrate (unanchored NEDD8 chains). Unanchored ubiquitin chains have been reported to control several processes, including innate immune response, viral uncoating, and aggresome formation (Banerjee et al., 2014; Hao et al., 2013; Ouyang et al., 2012; Zeng et al., 2010). The presented biochemical data on the role of unanchored NEDD8 chains on HSP70 function along with recent studies proposing substrate independent functions for NEDD8 in the control of poly(ADP-ribose) polymerase 1 (PARP-1) activity (Keuss et al., 2019) suggest that unanchored NEDD8 chains can act as regulatory signals.

The observed downregulation of NEDP1 levels in HCC indicates that NEDP1 is a target during tumorigenesis. This finding defines a molecular basis for the reported defects in the NEDD8 cycle in tumors as evidenced by an increase in protein NEDDylation (Abidi and Xirodimas, 2015). As inhibitors for the NEDD8 pathway are in phase II clinical trials (Abidi and Xirodimas, 2015), NEDP1 levels may provide a marker for the tumor response to these inhibitors.

STAR★METHODS

Detailed methods are provided in the online version of this paper and include the following:

- KEY RESOURCES TABLE
- LEAD CONTACT AND MATERIALS AVAILABILITY
- EXPERIMENTAL MODEL AND SUBJECT DETAILS
  - ○ Worm Strain and Culture Conditions
  - ○ Cell Culture
  - ○ Mouse Model Systems
- METHOD DETAILS
  - ○ Worm Protein Extraction and Fractionation
- STABLE ISOTOPE LABELING WITH AMINO ACID IN NEMATODES (SILAC)
  - ○ Worm Isotope Labeling
  - ○ Mass Spectrometry
  - ○ Worm Live Imaging Preparation
  - ○ Fluorescence Recovery after Photobleaching (FRAP)
  - ○ Scoring Cell Cycle Arrest in *C. Elegans*
  - ○ *C. Elegans* RNAi
  - ○ qRT-PCR Experiments
  - ○ ULP-3 Antibody Production
  - ○ ULP-3 and NEDD8 Site-Directed Mutagenesis
  - ○ Real Time PCR-Human Cells/Mice
  - ○ Protein Extraction from Liver Tissue
  - ○ Tissue Culture and siRNA/Plasmid Transfections
  - ○ Stable Cell Lines
  - ○ Isolation of His_6-NEDDylated/Ubiquitinated Proteins
  - ○ Caspase 3/7 Assay-Annexin V Staining
  - ○ CRISPR/Cas9 Deletion of NEDP1
  - ○ Subcellular Fractionation
  - ○ Immunoprecipitations
  - ○ *In Vitro* NEDD8 Processing Assay
  - ○ Sucrose Gradient Fractionation
  - ○ Purification of Recombinant His_6-NEDD8 Constructs
  - ○ Purification of Recombinant GST HSP70 Constructs
  - ○ Measurements of HSP70 ATPase Activity
  - ○ Isolation of Poly-NEDD8 Conjugates from Cell Extracts and ATPase Activity Measurement
  - ○ *In Vitro* Binding Assay
  - ○ *In Vitro* NEDDylation Assay
  - ○ GST Pull-Down from Cell Extracts
- QUANTIFICATION AND STATISTICAL ANALYSIS
- DATA AND CODE AVAILABILITY

SUPPLEMENTAL INFORMATION

Supplemental Information can be found online at https://doi.org/10.1016/j.celrep.2019.08.070.

ACKNOWLEDGMENTS

We are grateful to the Montpellier RIO Imaging facility. The project is funded by an “ATIP/AVENIR” fellowship; the Labex EpiGenMed; an “Investissements d’avenir” program, reference ANR-10-LABEX-12-01; INCa (PLBIO16-251); the Marie Curie FP7 Re-integration program; the Ministerio de Economía, Industria y Competitividad: SAF2017-87301-R (M.L.M.-C.); the Spanish Association against Cancer “Rare Cancer” (M.L.M.-C.); and the Wellcome Trust (AG 0909444/Z/09/Z). A.P.B. is a recipient of an “ANR retour” postdoctoral fellowship, and M.S.-M. is a recipient of an Asociación Española contra el Cáncer predoctoral fellowship.

AUTHOR CONTRIBUTIONS

A.P.B. and A.P. performed all experiments in *C. elegans*. A.P.B. performed the biochemical analysis on HSP70-NEDD8 interaction. H.T., C.M., and O.L. performed the experiments in human cells. M.S.-M. designed and performed all experiments in mice. D.P.X., A.G., and A.P.B. conceived and designed the experiments. D.P.X., with the help of all authors, wrote the manuscript.

DECLARATION OF INTERESTS

The authors declare no competing interests.

Received: March 15, 2019
Revised: June 27, 2019
Accepted: August 22, 2019
Published: October 1, 2019

REFERENCES

Abidi, N., and Xirodimas, D.P. (2015). Regulation of cancer-related pathways by protein NEDDylation and strategies for the use of NEDD8 inhibitors in the clinic. Endocr. Relat. Cancer *22*, T55–T70.

Ahmad, A., Bhattacharya, A., McDonald, R.A., Cordes, M., Ellington, B., Bertelsen, E.B., and Zuiderweg, E.R.P. (2011). Heat shock protein 70 kDa chaperone/DnaJ cochaperone complex employs an unusual dynamic interface. Proc. Natl. Acad. Sci. USA *108*, 18966–18971.

Aoki, I., Higuchi, M., and Gotoh, Y. (2013). NEDDylation controls the target specificity of E2F1 and apoptosis induction. Oncogene *32*, 3954–3964.

Bailly, A., and Gartner, A. (2013). Germ cell apoptosis and DNA damage responses. Adv. Exp. Med. Biol. *757*, 249–276.

Bailly, A.P., Freeman, A., Hall, J., Déclais, A.-C., Alpi, A., Lilley, D.M.J., Ahmed, S., and Gartner, A. (2010). The Caenorhabditis elegans homolog of Gen1/Yen1 resolvases links DNA damage signaling to DNA double-strand break repair. PLoS Genet. *6*, e1001025.

Banerjee, I., Miyake, Y., Nobs, S.P., Schneider, C., Horvath, P., Kopf, M., Matthias, P., Helenius, A., and Yamauchi, Y. (2014). Influenza A virus uses the aggresome processing machinery for host cell entry. Science *346*, 473–477.

Barbier-Torres, L., Delgado, T.C., García-Rodríguez, J.L., Zubiete-Franco, I., Fernández-Ramos, D., Buqué, X., Cano, A., Gutiérrez-de Juan, V., Fernández-Domínguez, I., Lopitz-Otsoa, F., et al. (2015). Stabilization of LKB1 and Akt by neddylation regulates energy metabolism in liver cancer. Oncotarget *6*, 2509–2523.

Beere, H.M., Wolf, B.B., Cain, K., Mosser, D.D., Mahboubi, A., Kuwana, T., Tailor, P., Morimoto, R.I., Cohen, G.M., and Green, D.R. (2000). Heat-shock protein 70 inhibits apoptosis by preventing recruitment of procaspase-9 to the Apaf-1 apoptosome. Nat. Cell Biol. *2*, 469–475.

Bratton, S.B., and Salvesen, G.S. (2010). Regulation of the Apaf-1-caspase-9 apoptosome. J. Cell Sci. *123*, 3209–3214.

Brenner, S. (1974). The genetics of Caenorhabditis elegans. Genetics *77*, 71–94.

Broemer, M., Tenev, T., Rigbolt, K.T.G., Hempel, S., Blagoev, B., Silke, J., Ditzel, M., and Meier, P. (2010). Systematic in vivo RNAi analysis identifies IAPs as NEDD8-E3 ligases. Mol. Cell *40*, 810–822.

Cain, K., Brown, D.G., Langlais, C., and Cohen, G.M. (1999). Caspase activation involves the formation of the aposome, a large (approximately 700 kDa) caspase-activating complex. J. Biol. Chem. *274*, 22686–22692.

Christmann, M., Schmaler, T., Gordon, C., Huang, X., Bayram, O., Schinke, J., Stumpf, S., Dubiel, W., and Braus, G.H. (2013). Control of multicellular development by the physically interacting deneddylases DEN1/DenA and COP9 signalosome. PLoS Genet. *9*, e1003275.

Coleman, K.E., Békés, M., Chapman, J.R., Crist, S.B., Jones, M.J., Ueberheide, B.M., and Huang, T.T. (2017). SENP8 limits aberrant neddylation of NEDD8 pathway components to promote cullin-RING ubiquitin ligase function. eLife *6*, e24325.

Delgado, T.C., Barbier-Torres, L., Zubiete-Franco, I., Lopitz-Otsoa, F., Varela-Rey, M., Fernández-Ramos, D., and Martínez-Chantar, M.-L. (2018). Neddylation, a novel paradigm in liver cancer. Transl. Gastroenterol. Hepatol. *3*, 37.

Enchev, R.I., Schulman, B.A., and Peter, M. (2015). Protein neddylation: beyond cullin-RING ligases. Nat. Rev. Mol. Cell Biol. *16*, 30–44.

Guan, J., Yu, S., and Zheng, X. (2018). NEDDylation antagonizes ubiquitination of proliferating cell nuclear antigen and regulates the recruitment of polymerase η in response to oxidative DNA damage. Protein Cell *9*, 365–379.

Hao, R., Nanduri, P., Rao, Y., Panichelli, R.S., Ito, A., Yoshida, M., and Yao, T.-P. (2013). Proteasomes activate aggresome disassembly and clearance by producing unanchored ubiquitin chains. Mol. Cell *51*, 819–828.

Hjerpe, R., Thomas, Y., Chen, J., Zemla, A., Curran, S., Shpiro, N., Dick, L.R., and Kurz, T. (2012). Changes in the ratio of free NEDD8 to ubiquitin triggers NEDDylation by ubiquitin enzymes. Biochem. J. *441*, 927–936.

Jackson, S.P., and Durocher, D. (2013). Regulation of DNA damage responses by ubiquitin and SUMO. Mol. Cell *49*, 795–807.

Jiang, J., Maes, E.G., Taylor, A.B., Wang, L., Hinck, A.P., Lafer, E.M., and Sousa, R. (2007). Structural basis of J cochaperone binding and regulation of Hsp70. Mol. Cell *28*, 422–433.

Jin, J., Ang, X.L., Shirogane, T., and Wade Harper, J. (2005). Identification of dubstrates for F-Box proteins. In Methods in Enzymology, R.J. Deshaies, ed. (Elsevier), pp. 287–309.

Kessler, B.M. (2013). Ubiquitin - omics reveals novel networks and associations with human disease. Curr. Opin. Chem. Biol. *17*, 59–65.

Keuss, M.J., Hjerpe, R., Hsia, O., Gourlay, R., Burchmore, R., Trost, M., and Kurz, T. (2019). Unanchored tri-NEDD8 inhibits PARP-1 to protect from oxidative stress-induced cell death. EMBO J. *38*, e100024.

Kim, W., Bennett, E.J., Huttlin, E.L., Guo, A., Li, J., Possemato, A., Sowa, M.E., Rad, R., Rush, J., Comb, M.J., et al. (2011). Systematic and quantitative assessment of the ubiquitin-modified proteome. Mol. Cell *44*, 325–340.

Kityk, R., Kopp, J., Sinning, I., and Mayer, M.P. (2012). Structure and dynamics of the ATP-bound open conformation of Hsp70 chaperones. Mol. Cell *48*, 863–874.

Larance, M., Bailly, A.P., Pourkarimi, E., Hay, R.T., Buchanan, G., Coulthurst, S., Xirodimas, D.P., Gartner, A., and Lamond, A.I. (2011). Stable-isotope labeling with amino acids in nematodes. Nat. Methods *8*, 849–851.

Leidecker, O., Matic, I., Mahata, B., Pion, E., and Xirodimas, D.P. (2012). The ubiquitin E1 enzyme Ube1 mediates NEDD8 activation under diverse stress conditions. Cell Cycle *11*, 1142–1150.

Lettre, G., and Hengartner, M.O. (2006). Developmental apoptosis in C. elegans: a complex CEDnario. Nat. Rev. Mol. Cell Biol. *7*, 97–108.

Li, T., Guan, J., Huang, Z., Hu, X., and Zheng, X. (2014). RNF168-mediated H2A neddylation antagonizes ubiquitylation of H2A and regulates DNA damage repair. J. Cell Sci. *127*, 2238–2248.

Liu, G., and Xirodimas, D.P. (2010). NUB1 promotes cytoplasmic localization of p53 through cooperation of the NEDD8 and ubiquitin pathways. Oncogene *29*, 2252–2261.

Ma, T., Chen, Y., Zhang, F., Yang, C.-Y., Wang, S., and Yu, X. (2013). RNF111-dependent neddylation activates DNA damage-induced ubiquitination. Mol. Cell *49*, 897–907.

Maghames, C.M., Lobato-Gil, S., Perrin, A., Trauchessec, H., Rodriguez, M.S., Urbach, S., Marin, P., and Xirodimas, D.P. (2018). NEDDylation promotes nuclear protein aggregation and protects the Ubiquitin Proteasome System upon proteotoxic stress. Nat. Commun. *9*, 4376.

Mahata, B., Sundqvist, A., and Xirodimas, D.P. (2012). Recruitment of RPL11 at promoter sites of p53-regulated genes upon nucleolar stress through NEDD8 and in an Mdm2-dependent manner. Oncogene *31*, 3060–3071.

Martínez-Chantar, M.L., Vázquez-Chantada, M., Ariz, U., Martínez, N., Varela, M., Luka, Z., Capdevila, A., Rodríguez, J., Aransay, A.M., Matthiesen, R., et al. (2008). Loss of the glycine N-methyltransferase gene leads to steatosis and hepatocellular carcinoma in mice. Hepatology *47*, 1191–1199.

Martínez-López, N., García-Rodríguez, J.L., Varela-Rey, M., Gutiérrez, V., Fernández-Ramos, D., Beraza, N., Aransay, A.M., Schlangen, K., Lozano, J.J., Aspichueta, P., et al. (2012). Hepatoma cells from mice deficient in glycine N-methyltransferase have increased RAS signaling and activation of liver kinase B1. Gastroenterology *143*, 787–798.e13.

Mayer, M.P., and Bukau, B. (2005). Hsp70 chaperones: cellular functions and molecular mechanism. Cell. Mol. Life Sci. *62*, 670–684.

Mergner, J., Heinzlmeir, S., Kuster, B., and Schwechheimer, C. (2015). DENEDDYLASE1 deconjugates NEDD8 from non-cullin protein substrates in *Arabidopsis thaliana*. Plant Cell *27*, 741–753.

Mergner, J., Kuster, B., and Schwechheimer, C. (2017). DENEDDYLASE1 Protein Counters Automodification of Neddylating Enzymes to Maintain NEDD8 Protein Homeostasis in *Arabidopsis*. J. Biol. Chem. *292*, 3854–3865.

Ordureau, A., Münch, C., and Harper, J.W. (2015). Quantifying ubiquitin signaling. Mol. Cell *58*, 660–676.

Ouyang, H., Ali, Y.O., Ravichandran, M., Dong, A., Qiu, W., MacKenzie, F., Dhe-Paganon, S., Arrowsmith, C.H., and Zhai, R.G. (2012). Protein aggregates are recruited to aggresome by histone deacetylase 6 via unanchored ubiquitin C termini. J. Biol. Chem. *287*, 2317–2327.

Palleros, D.R., Welch, W.J., and Fink, A.L. (1991). Interaction of hsp70 with unfolded proteins: effects of temperature and nucleotides on the kinetics of binding. Proc. Natl. Acad. Sci. USA *88*, 5719–5723.

Popovic, D., Vucic, D., and Dikic, I. (2014). Ubiquitination in disease pathogenesis and treatment. Nat. Med. *20*, 1242–1253.

Qi, S., Pang, Y., Hu, Q., Liu, Q., Li, H., Zhou, Y., He, T., Liang, Q., Liu, Y., Yuan, X., et al. (2010). Crystal structure of the Caenorhabditis elegans apoptosome reveals an octameric assembly of CED-4. Cell *141*, 446–457.

Rodriguez, J., and Lazebnik, Y. (1999). Caspase-9 and APAF-1 form an active holoenzyme. Genes Dev. *13*, 3179–3184.

Saleh, A., Srinivasula, S.M., Balkir, L., Robbins, P.D., and Alnemri, E.S. (2000). Negative regulation of the Apaf-1 apoptosome by Hsp70. Nat. Cell Biol. *2*, 476–483.

Shen, L.N., Liu, H., Dong, C., Xirodimas, D., Naismith, J.H., and Hay, R.T. (2005). Structural basis of NEDD8 ubiquitin discrimination by the deNEDDylating enzyme NEDP1. EMBO J. *24*, 1341–1351.

Sui, Y., Liu, Y., and Xu, G. (2015). A lysine-to-arginine mutation on NEDD8 markedly reduces the activity of cullin RING E3 ligase through the impairment of neddylation cascades. Biochem. Biophys. Res. Commun. *461*, 653–658.

Sundqvist, A., Liu, G., Mirsaliotis, A., and Xirodimas, D.P. (2009). Regulation of nucleolar signalling to p53 through NEDDylation of L11. EMBO Rep. *10*, 1132–1139.

Tatham, M.H., Rodriguez, M.S., Xirodimas, D.P., and Hay, R.T. (2009). Detection of protein SUMOylation in vivo. Nat. Protoc. *4*, 1363–1371.

Watson, I.R., Li, B.K., Roche, O., Blanch, A., Ohh, M., and Irwin, M.S. (2010). Chemotherapy induces NEDP1-mediated destabilization of MDM2. Oncogene *29*, 297–304.

Williamson, A., Werner, A., and Rape, M. (2013). The Colossus of ubiquitylation: decrypting a cellular code. Mol. Cell *49*, 591–600.

Wolter, K.G., Hsu, Y.T., Smith, C.L., Nechushtan, A., Xi, X.G., and Youle, R.J. (1997). Movement of Bax from the cytosol to mitochondria during apoptosis. J. Cell Biol. *139*, 1281–1292.

Xirodimas, D., Saville, M.K., Edling, C., Lane, D.P., and Laín, S. (2001). Different effects of p14ARF on the levels of ubiquitinated p53 and Mdm2 in vivo. Oncogene *20*, 4972–4983.

Xirodimas, D.P., Sundqvist, A., Nakamura, A., Shen, L., Botting, C., and Hay, R.T. (2008). Ribosomal proteins are targets for the NEDD8 pathway. EMBO Rep. *9*, 280–286.

Xu, G., Paige, J.S., and Jaffrey, S.R. (2010). Global analysis of lysine ubiquitination by ubiquitin remnant immunoaffinity profiling. Nat. Biotechnol. *28*, 868–873.

Young, J.C. (2010). Mechanisms of the Hsp70 chaperone system. Biochem. Cell Biol. *88*, 291–300.

Zeng, W., Sun, L., Jiang, X., Chen, X., Hou, F., Adhikari, A., Xu, M., and Chen, Z.J. (2010). Reconstitution of the RIG-I pathway reveals a signaling role of unanchored polyubiquitin chains in innate immunity. Cell *141*, 315–330.

Zhao, Y., Brickner, J.R., Majid, M.C., and Mosammaparast, N. (2014). Crosstalk between ubiquitin and other post-translational modifications on chromatin during double-strand break repair. Trends Cell Biol. *24*, 426–434.

Zhuravleva, A., Clerico, E.M., and Gierasch, L.M. (2012). An interdomain energetic tug-of-war creates the allosterically active state in Hsp70 molecular chaperones. Cell *151*, 1296–1307.

STAR★METHODS

KEY RESOURCES TABLE

REAGENT or RESOURCE	SOURCE	IDENTIFIER
Antibodies		
Rabbit monoclonal anti-NEDD8, Y297	GeneTex	Cat#GTX61205; RRID:AB_10619223
Rabbit anti-ubiquitin, western blotting	DAKO	Cat#z0458; RRID:AB_2315524
Mouse anti- β-actin	Calbiochem	Ab-1
Rabbit anti-APAF-1, E38 (IPs)	Abcam	Cat# ab32372, RRID:AB_722822
Mouse anti-APAF-1 (input)	BD Biosciences	Cat#611364; RRID:AB_2056905
Mouse anti-GAPDH (C65)	Abcam	Cat#Ab8245; RRID:AB_2107448
Rabbit anti-UBC12	Abgent	Cat#AP2169b; RRID:AB_353056
Mouse anti-cullin 1	Zymed	Cat#32-2400; RRID:AB_2533070
Rabbit anti-cullin 2	Abcam	Cat#Ab187504
Rabbit anti-cullin 3	Cell Signaling	Cat#2759S; RRID:AB_2086432
Rabbit anti-cullin 4A	Abcam	Cat#Ab34897; RRID:AB_731766
Sheep anti-cullin 5	Dr Arno Alpi	N/A
Rabbit anti-H2Ax	Abcam	Cat#Ab11175; RRID:AB_297814
Rabbit anti-γH2Ax	Millipore	Cat#05-636; RRID:AB_309864
Mouse anti-tubulin	Cell Signaling	Cat#3873; RRID:AB_1904178
Rabbit anti-H2A	Abcam	Cat#Ab13923; RRID:AB_300750
Mouse anti-HSP70	Abcam	Cat#Ab47455; RRID:AB_881520
Mouse anti-p53 DO-1	In house	N/A
Sheep anti-NEDP1	In house	N/A
Rabbit anti-ULP-3	In house	N/A
Mouse Flag and anti-Flag M2 affinity gel	SIGMA	Cat#A2220; RRID:AB_10063035
Ubiquitin Branch Motif Antibody (K-ε-GG)	Cell Signaling	Cat#3925
Chemicals, Peptides, and Recombinant Proteins		
MLN4924	Active Biochem	A-1139
Lipofectamine RNAiMAX	Invitrogen	13778150
4-12% Bis-Tris gels	Invitrogen	NP0322
siRNA On-TARGETplus SMARTpools	Dharmacon	N/A
Compounds	Sigma Aldrich	N/A
Fugene6 HD	Roche	11814443001
Protease Inhibitor Cocktail Tablets EDTA-free	Roche	11873580001
Ni-NTA Agarose	QIAGEN	30210
DNAase I	Invitrogen	18068015
Amylose resin	New England Biolabs	E8021S
Cobalt Talon beads	Clontech	635501
AffiGel-15 resin	BioRad	**1536051**
Annexin V	BD Biosciences	556547
Thyroglobulin	BioRad	PHP236
PVDF membrane	Millipore	IPVH00010
ECL Western Blotting Detection Reagents	Amersham	RPN2106
Protein G Sepharose beads	Amersham	17061801
Medical X-ray films	Konica	A9KN
Labeled amino acids	Euriso-Top	N/A

(*Continued on next page*)

Continued

REAGENT or RESOURCE	SOURCE	IDENTIFIER
**Critical Commercial Assays**		
RNeasy® Kit	QIAGEN	74104
QuantiTech® Reverse Transcription Kit	QIAGEN	205310
qPCR Master Mix	Eurogentec	05-QP2X-03
SYBR™ Select Master Mix	Applied Biosystems	44-729-08
SV Total RNA Isolation kit	Promega	Z3101
SuperScript III First-Strand Synthesis SuperMix	Invitrogen	18080400
CellTiter-Glo® Luminescent Cell Viability Assay	Promega	G7570
Caspase-Glo 3/7 Assay	Promega	G8091
Green malachite reagent	SIGMA	38800
**Deposited Data**		
SILAC Proteomics data	MassIVE	MSV000084214
**Experimental Models: Cell Lines**		
Human: U2OS	ATCC	HTB-96
Human: MCF7	ATCC	HTB-22
Human: H1299	ATCC	CRL-5803
Mouse: OKER cells	Labortory of Martinez-Chantar	Martínez-López et al., 2012
**Experimental Models: Organisms/Strains**		
C. elegans strains: *ced-1(e1935), ced-3(n717), ced-9(n1653ts), cep-1(lg12501), gla-3(op216), glp-4(bn2ts), hpr-17(tm1579), lig-4(ok716), opls219* (CED-4::GFP), *orn-1(tm5454), ulp-3 (tm1287).* The *ulp-3(tm1287)* and *orn-1(tm5454)*	National Bioresourse Project for the Expremental Animal "Nematode C elegans" (NBRP)	N/A
GNMT-KO male mice	Martinez-Chantar laboratory	N/A
**Oligonucleotides**		
RNAi oligos C. elegans ulp-3: F:5′- TATATATAGCGGCCGCATGTCAGTTCCCCCGG	This paper	N/A
RNAi oligos C. elegans ned-8: F:5′-AGGCGGCCGCTTAAAATCCTCCGCGG	This paper	N/A
PCR ulp-3 primers: F:5′-TATATATAGGCGCGCCATGTCAGTTCCCCC GGATTTCCGC	This paper	N/A
Mice qPCR primers, nedp1 F:5′-ATCCCTGCTCAATACAAGATGGA	This paper	N/A
Mice qPCR primers, gapdh F:5′GGATGCAGGGATGATGTTC	This paper	N/A
**Recombinant DNA**		
pcDNA3 Flag-RPL11	Sundqvist et al., 2009	N/A
ULP-3 WT and mutants	This paper	N/A
His_6-NEDD8 WT	Xirodimas et al., 2001	N/A
His_6-NEDD8 K11/48R	This paper	N/A
His_6-NEDD8 ΔG	This paper	N/A
His_6-NEDD8 KR mutants	This paper	N/A
His_6-ubiquitin	Xirodimas et al., 2001	N/A
GST-HSP70 full-length and mutants	This paper	N/A
GST-HSP40	This paper	N/A
NEDP1 wild type, C163A	Shen et al., 2005	N/A
pcDNA3-DN-hCUL5-Flag	Jin et al., 2005	Addgene plasmid #15823

(*Continued on next page*)

Continued		
REAGENT or RESOURCE	SOURCE	IDENTIFIER
Software and Algorithms		
Leica LAS		https://www.leica-microsystems.com/
Image Gauge		https://www.fujifilm.com
SEQUEST 3G and the SORCERER 2		v4.0, Milpitas CA

LEAD CONTACT AND MATERIALS AVAILABILITY

Further information and requests for resources and reagents should be directed to and will be fulfilled by the Lead Contact, Dimitris P. Xirodimas (dimitris.xirodimas@crbm.cnrs.fr). Plasmids newly generated in this study will be made readily available to the scientific community. We will honor requests in a timely fashion.

EXPERIMENTAL MODEL AND SUBJECT DETAILS

Worm Strain and Culture Conditions

Hermaphrodite worms were maintained at 20°C on NGM agar plates according to standard procedure unless otherwise indicated (Brenner, 1974). The following mutations and transgenes used in this study were: *ced-1(e1935), ced-3(n717), ced-9(n1653ts), cep-1(lg12501), gla-3(op216), glp-4(bn2ts), hpr-17(tm1579), lig-4(ok716), opIs219* (CED-4::GFP), *orn-1(tm5454), ulp-3(tm1287).* The *ulp-3(tm1287)* and *orn-1(tm5454)* deletion alleles were obtained from the Japanese Consortium (NBRP) and backcrossed five times against our N2 wild-type genetic background. Double and triple mutants generated in this study will be available at the CGC and/or available on request. Apoptosis, cell cycle arrest assays and genotoxic sensitivity testing were performed as previously described (Bailly et al., 2010; Bailly and Gartner, 2013).

Cell Culture

Cell lines, U2OS (Female), MCF7 (Female), H1299 (Male) were originally obtained from the ATCC bioresource. Hepatoma cell line derived from GNMT-KO mice liver tumors (OKER cells) (Martínez-López et al., 2012), were employed for the experiment described in Figure 7D. Cell lines were maintained in DMEM with the exception of H1299 (RPMI), in 10% FCS and standard antibiotics, in 5% CO2 and 37°C and regularly tested for mycoplasma contamination. Cell lines have not been authenticated.

Mouse Model Systems

Animal experiments were performed according to the Spanish Guide for Care and Use of laboratory animals and the European Research Council animal care and use guidelines. Protocols were approved by the CIC bioGUNE Animal Care and Use Committee and the local authority (Diputación de Bizkaia). In the present study, animals were between 5 to 12-month-old males. Animals were bred and housed in the animal unit of CIC bioGUNE (AAALAC-accredited facility) with controlled conditions of temperature (22°C) and humidity, in a 12 h light/dark cycle with *ad libitum* access to food and water.

METHOD DETAILS

Worm Protein Extraction and Fractionation

For Western Blots, worm proteins were extracted from synchronized adult worms in 50mM HEPES-NaOH pH8.0, Urea 9M, SDS 2% supplemented with zirconium 0.7mm beads (BioSpec Product) and disrupted in a bead beater (FastPrep, 3 cycles of 6 m/sec, 20sec each). Debris were removed by quick centrifugation cycles (1000 g, 1min, 3 times) and soluble proteins were then separated by a 12000 g, 30min centrifugation. Supernatant protein concentration was measured by BCA following manufacturer's instructions (Life technologies). 2xSDS Laemmli buffer was added before boiling samples for 5min.

STABLE ISOTOPE LABELING WITH AMINO ACID IN NEMATODES (SILAC)

Worm Isotope Labeling

To minimize Arginine-to-Proline conversion we obtained a deletion allele from the Japanese NBRP consortium that disrupts the *orn-1* gene *(tm5454)*. We backcrossed the *orn-1(tm5454)* deletion five times against our N2 wild-type background then against *ulp-3(tm1287).* For isotopic labeling, SILAC bacteria (SLE1) were labeled as in Larance et al. (2011). Synchronized worms were grown on nitrogen free nematode growth medium (NGM-N) as described previously (Larance et al., 2011) seeded with SLE1 bacteria labeled with Light (K0R0) and Heavy (K8R10) Isotopes (Eurisotope). Larvae stage 1 synchronized worms were grown until late adult stage and synchronized again by bleaching before growing them on 9cm NGM-N plates seeded with SILAC bacteria (SLE1). Approximately 10 plates of 4000 worms each were used to grow synchronized and labeled worms until late L4-young adult stage that were

irradiated with 90Gy of IR using a Blood-Xrad apparatus. Labeled worms proteins were extracted in 50mM HEPES-NaOH pH8.0, Urea 9M using a bead beater and zirconium 0.7mm beads following the same procedure as described in the protein extraction section.

Mass Spectrometry

Di-Glycine motif peptide identification was performed by Cell Signaling Technology, following UbiScan protocols and instructions (Cell Signaling Technology) using Ubiquitin Branch Motif Antibody (K-ε-GG) #3925. Peptides were loaded onto 10cm x 75 μm PicoFrit Capillary column packed with Magic C18 AQ reversed-phase resin. The column was developed with a 90min linear gradient of acetonitrile in 0.125% formic acid delivered at 280nL/min. MS parameters settings: MS run time 96min, MS1 Scan Range (300.0-1500.00), Top 20 MS/MS (Min Signal 500, isolation width 2.0, normalized coll. energy 35.0, activation-Q 0.250, activation time 20.0, lock mass 371.101237, charge state rejection enabled, charge state 1+ rejected, dynamic exclusion enabled, repeat count 1, repeat duration 35.0, exclusion list size 500, exclusion duration 40.0, exclusion mass width relative to mass, exclusion mass width 10 ppm). MS/MS spectra were evaluated using SEQUEST 3G and the SORCERER 2 platform from Sage-N Research (v4.0, Milpitas CA). Searches were performed against the most recent update of the NCBI *C. elegans* database with mass accuracy of ± 50ppm for precursor ions and 1Da for product ions. Results were filtered with mass accuracy of ± 5 ppm on precursor ions and presence of the intended motif (K-ε-GG). LTQ-Orbitrap Velos was used (Thermoscientific). A 5% default false positive rate was used to filter the SORCERER results.

Worm Live Imaging Preparation

For FRAP-based acquisitions, worms were individually picked on an unseeded NGM plate to wash off bacteria and transferred on a glass slide in a 10 μL drop of egg buffer (118mM NaCl, 48mM KCl, 2mM $CaCl_2$*$2H_2O$, 2mM $MgCl_2$*$6H_2O$, 25mM HEPES pH 7.3). Worm gonads were dissected with a 23G syringe and immediately covered with a coverslip sealed with nail polish. Acquisitions were performed immediately after (less than 5min).

Fluorescence Recovery after Photobleaching (FRAP)

FRAP analysis was performed on a confocal microscope Leica Sp5, objective Leica 63x/1.4 oil Apo. GFP was photo-bleached by "FRAP wizard" function of Leica LAS software. The region of one nucleus was photo-bleached with maximal 488nm laser to obtain more than 70% of photo-bleaching and recovery with 5% of laser power. Fluorescence intensities were measured by five acquisitions in pre-bleached and twenty acquisitions every 0.34sec (post–bleached 1) and every 10sec nine times during the recovery (post-bleached 2). Intensities data of bleach, background and non-bleached nucleus regions (total intensity) were extracted from the Leica software and analyzed with excel to calculate fluorescent protein mobility. The fraction of the fluorescent protein that was mobile was calculated by comparing the fluorescence intensities of non-bleached and bleached nucleus.

Scoring Cell Cycle Arrest in *C. Elegans*

Cell cycle arrest was scored as described in Bailly et al. (2010). Briefly, at least ten worm germlines are dissected and stained with DAPI; all mitotic cells within 50 μm from the distal tip cell are counted and a statistical analysis is presented (n = 10, ± SEM).

C. Elegans RNAi

We adapted the RNAi bacterial feeding method from Larance et al. (2011). Briefly, *E. coli* HT115 RNAi bacteria were grown in M9 minimal supplemented with glucose 0.4% (wt/vol), 2mM MgSO4, 0.1mM $CaCl_2$, thiamine 0.01% (wt/vol) and 100 μg/ml ampicillin. A single bacteria colony streaked on LB plate was used to start the culture at 37°C under agitation until OD600nm = 1. Double-stranded RNA expression was induced by adding IPTG to a final concentration of 0.5mM for 3hrs. Bacteria culture was seeded on NGM plates complemented with ampicillin. Worms were placed on NGM plates seeded with RNAi bacteria at late L1 stage for *ulp-3* RNAi and L3 stage for *ned-8* RNAi. Late L4 to young adult stage worms were analyzed. We found that growing HT115 bacteria in minimal medium greatly increases RNAi efficiency even for loci reported to be problematic to achieve appropriate depletion level.

For ul*p-3* and *ned-8* RNAi, the following oligos were used to clone cDNA into the L4440 vector:

For *ulp-3*:
5′ to 3′: TATATATAGCGGCCGCATGTCAGTTCCCCCGG
5′ to 3′: TATATATAGCGGCCGCTTATTTCGCTTCAAAATTAACAATC
For *ned-8*:
5′ to 3′: AGGCGGCCGCTTAAAATCCTCCGCGG
5′ to 3′: AAGGCGGCCGCATGCTCATCAAAGTTA

cDNA were prepared as described in the qRT-PCR section.

qRT-PCR Experiments

Gravid adult worms were collected from the plates and washed three times to remove bacteria with PBS before introduction in Trizol and frozen at -80°C. Total RNA was extracted by Trizol/chloroform followed by RNeasy® Kit (QIAGEN) and cDNA synthesis performed on 300ng of RNA with QuantiTech® Reverse Transcription Kit (QIAGEN). qRT-PCR reactions were performed in Roche LC480 qPCR in triplicates, and cDNA was diluted in Mesa Green qPCR Master Mix (Eurogentec) with specific primers. The mean values for the levels of transcript of interest (*egl-1*) were normalized with internal level of control transcript (tubulin *tbg-1*) relatively stably expressed between all experiments. For data analysis, maximum quantitative point method was applied and induction was calculated with the equation:

$$\text{Fold Induction} = 2^{\wedge}\left(\left(^{\Delta Cp(cDNAegl-1-cDNAbg-)}\text{control}\right) - \left(^{\Delta Cp(cDNAegl-1-cDNAtbg-)}\text{treated}\right)\right).\text{Data represent the average} + / - \text{SEM}$$

ULP-3 Antibody Production

Full length ulp-3 was amplified from cDNA derived from adult worms using the same protocol as used in qRT-PCR experiments section using the following oligo:

5′-TATATATAGGCGCGCCATGTCAGTTCCCCCGGATTTCCGC (forward)
5′-GAAGCGAAATAAGGCCGGCCTATATATA (reverse)

AscI was introduced at the 5′ of the start codon and FseI at the 3′ of the stop codon and cloned into appropriately modified pQE-80L (His_6) and pMAL-c2 (MBP). Protein expression of MBP-ULP-3 and His_6-ULP-3 was performed in BL21(DE3) CodonPlus bacteria grown and induced at 37°C for 2hrs with 0.5mM IPTG. MBP-ULP-3 was purified according to manufacturer standard protocols for amylose resin (New England Biolabs). His_6-ULP-3 was purified on Cobalt Talon beads (Clontech) following manufacturer instructions for soluble protein and finally dialysed three times against 50mM Tris-HCl pH7.5, 150mM NaCl, 10% glycerol, 0.5mM DTT. MBP-ULP-3 was used to immunize rabbits (BioGenes GmbH). For affinity purification of the antibodies, His_6-ULP-3 was covalently coupled to AffiGel-15 resin (BioRad) and used to purify final bleeds serum.

ULP-3 and NEDD8 Site-Directed Mutagenesis

The catalytically inactive mutant of MBP-ULP-3 (C167A), the hydrophobic patch (I44A) NEDD8 mutant and all KR NEDD8 mutants were generated by site directed mutagenesis and sequences were verified by automated sequencing.

Real Time PCR-Human Cells/Mice

RNA from cells in 6cm dishes was isolated with the Promega SV Total RNA Isolation kit. cDNA synthesis was performed using the Invitrogen SuperScript III First-Strand Synthesis SuperMix using 300ng of RNA. Real-time PCR was performed as described in Mahata et al. (2012) an ABI 7500 system using ABI PCR master mix. The TaqMan primers for p21 can be found in Mahata et al. (2012), whereas the nedp1 primers were purchased from ABI. RNA isolation from liver tissue was isolated using TRIZOL/Chloroform and RNA quantification was determined in the Nanodrop ND-100 spectrophotometer (ThermoFischer Scientifict, USA). 2μg of isolated RNA were treated with DNase I and cDNA was synthesized with M-MLV reverse transcriptase in the presence of random primers and RNaseOUT. Real time PCR was performed with 1.5 μL of cDNA (diluted 1:10), SYBR Select Master Mix. Primers for monitoring nedp1 (senp8) mRNA expression in mice: F: 5′ATCCCTGCTCAATACAAGATGGA, R: 5′ CAAACCCAATAATGTGGTCGTTG. Data were normalized against gapdh expression, F: 5′GGATGCAGGGATGATGTTC and R: 5′TGCACCACCAACTGCTTAG.

Protein Extraction from Liver Tissue

Approximately, 50μg of liver tissue were homogenized by using Precellys 24 (Bertin Technologies) with 1ml of lysis buffer (1.6mM NaH2PO4, 8.4mM Na2HPO4, 0.1% Triton X-100, 0.1M NaCl, 0.1% SDS, 0.5% sodium azide) supplemented with protease and phosphatase inhibitor cocktail, 20mM iodoacetamide and 20mM N-Ethylmaleimide. Lysates were centrifuged (13000rpm, 30min, 4°C) and total protein content in the supernatant was measured by Bradford. 20μg of protein was used for SDS-PAGE and western blot analysis.

Tissue Culture and siRNA/Plasmid Transfections

All cell lines were originally obtained from the ATCC bioresourse. Cell lines were maintained in DMEM in 10% FCS and standard antibiotics, in 5% CO2 and 37°C and regularly tested for mycoplasma contamination. For siRNA transfections 5nM of oligos were transfected with RNAiMAX lipofectamine. All siRNAs are from Dharmacon as ON-TARGET-Plus pools of 4 individual oligos. For the experiment in Figure 7D, 2 μg of plasmids were transfected in cells seeded in 6-well plates with Fugene6 HD.

Stable Cell Lines

U2OS cells stably expressing wild-type His_6-NEDD8 or His_6-NEDD8 K11/48R mutant was performed as described in Liu and Xirodimas (2010), using lentivirus with the respective constructs. Cells were selected and maintained in puromycin (2.5 μg/ml). This method provides clones expressing low levels of ectopic NEDD8 similar to the endogenous levels (Liu and Xirodimas, 2010). Several

assays (treatment with MLN4924, or proteotoxic stress) confirmed that the ectopic NEDD8 responds similarly to the endogenous NEDD8. Parental or H6 NEDP1 KO U2OS cells were transfected with either empty or 3xFlag-HSP70 pcDNA3 vectors. Cells were selected with G418 (1mg/ml) for 14 days before a pool of cells stably expressing 3xFlag-HSP70 was acquired.

Isolation of His_6-NEDDylated/Ubiquitinated Proteins

H1299 cells in 10cm dishes were transfected with 1 μg of His_6-NEDD8 or His_6-ubiquitin, 2 μg of Flag-L11, 3 μg of Flag-ULP-3 or NEDP1 with Fugene. 48hrs post transfection Ni^{2+}-pull downs and total cell extracts (input 2xSDS Laemmli buffer) were prepared as described in Tatham et al. (2009) and Xirodimas et al. (2001). For the experiments monitoring the conjugation of KR NEDD8 mutants, cells as indicated were transfected twice (24hrs apart with re-seeding) with NEDP1 siRNAs. In the first NEDP1 siRNA transfection, cells were also transfected with NEDD8 siRNA #07, which specifically targets the endogenous but not the ectopic NEDD8 (Sundqvist et al., 2009).

Caspase 3/7 Assay-Annexin V Staining

U2OS cells were transfected in 6 well plates. Next day cells were trypsinised and seeded in 24well plates (7000 cells/well) in duplicates. 24hrs later cells were treated as indicated and one set of cells was used to measure cell survival and the other caspase 3/7 activity. The ratio between caspase 3/7 activity over survival was measured and represented as fold change to the control untreated cells. For Annexin V staining 1x10cm 80% confluent U2OS cells was used for each condition. Cells were collected by careful trypsinisation (floating cells were also included), washed in PBS, before resuspended in binding buffer, $1x10^6$ cells/ml (10mM HEPES/NaOH, pH 7.4, 150mM NaCl, 5mM KCl, 5mM $MgCl_2$, 1.8mM $CaCl_2$) and FITC-Annexin V was added at 1:100 dilution. Samples were kept at RT in the dark for 20min before 7-ADD viability dye (5mg/sample, late apoptotic cells) was added and samples were analyzed by flow cytometry. For both assays data represent the average of 3 different experiments ± SEM.

CRISPR/Cas9 Deletion of NEDP1

The U6gRNA-Cas9-2A-GFP plasmid for NEDP1 was purchased from SIGMA. 2 plasmids with different guide sequences were obtained (#20, #22). U2OS cells were transfected in 10cm dishes with the plasmids and 24hrs later cells were collected and GFP positive cells were selected and separated in 96-well plates as single cells by flow cytometry. Several clones were screened for NEDP1 and NEDD8 expression. Total DNA from the used clone in this study (H6) was used to amplify the NEDP1 locus by PCR and sequencing of the PCR product confirmed the deletion in NEDP1 gene (305-314bp).

Subcellular Fractionation

2x10cm dishes of 80% confluent parental or H6 U2OS cells were used for fractionation. Cells were washed and collected in 1ml PBS and 100 μL of the sample were pelleted at 13000rpm for 1min and lysed with 100 μL of 2xSDS Laemmli buffer (input). The remaining 900 μL were pelleted at 10800rpm for 20 s and resuspended in 300 μL buffer A (10mM HEPES-KOH pH 8.0, 10mM KCl, 1.5mM $MgCl_2$) with protease inhibitor (complete EDTA-free, Roche) and 10mM iodoacetamide. Cells were lysed by adding Triton X-100 at a final concentration of 0.1% for 1min at 4°C, then centrifuged for 5min at 1300 g, 4°C. The supernatant (cytoplasmic fraction) was mixed with equal volume of 2xSDS Laemmli buffer. The pellet (nuclear fraction) was washed 3x with buffer A, then resuspended with 300 μL of buffer B (20mM HEPES-KOH pH 8.0, 300mM NaCl, 2mM EDTA and 1%NP40) and incubated 30min on ice. Lysates were centrifuged at 10800rpm for 20 s. The supernatant (nucleoplasmic fraction) was mixed with equal volume of 2xSDS Laemmli buffer. The pellet was washed 3 times with buffer B then resuspended in 600 μL of 2xSDS Laemmli buffer (pellet fraction).

Immunoprecipitations

For each condition 1x10cm dish of 80% confluent U2OS cells was used. Cells were washed 2x with PBS before collected and lysed in 50mM Tris-HCl, pH 7.4, 100mM KCl, 1% NP-40, 2mM EDTA, protease inhibitors (complete EDTA-free, Roche) for 15min on ice. Lysates were cleared by centrifugation at 13000rpm for 15min at 4°C and extracts of equal amount of total protein were used for immunoprecipitations overnight at 4°C with 2 μg of APAF1 antibody and 20 μL of protein G beads (prewashed with lysis buffer). Next day beads were washed 2x with 500 μL of lysis buffer (centrifuged at 5000rpm at 4°C in each wash), resuspended in 100 μL of 2xSDS Laemmli buffer and boiled for 5min. Immunoprecipitates were analyzed by western blotting. For Flag pull downs 20 μL of pre-washed Flag beads were used.

In Vitro NEDD8 Processing Assay

Protein induction in bacteria was performed with 0.5mM IPTG at 16°C overnight and induced proteins were purified as MBP fusions using standard methods. NEDP1 was purified as His_6-GST fusion before cleavage with thrombin as described in Shen et al. (2005). 600ng of MBP-NEDD8-ubiquitin fusion and 200ng of ULP-3/NEDP1 proteins were incubated for the indicated periods of time at 37°C in 10 μL of 50mM Tris, pH 7.5, 100mM NaCl, 5mM $MgCl_2$, 1mM DTT. Reactions were terminated with the addition of 10 μL of 2xSDS Laemmli buffer, samples were boiled for 5min, separated on a 4%–12% precast SDS-PAGE and Coomassie blue stained.

Sucrose Gradient Fractionation

4x10cm dishes of 80% confluent cells were washed 2x with PBS before collected and lysed in 50mM Tris, pH 7.4, 100mM KCl, 1% NP-40, 2mM EDTA, protease inhibitors (Roche tablets) for 15min on ice. After centrifugation at 14000rpm for 15min at 4°C, equal amount of extracts were loaded on 10%–50% sucrose gradient. Samples were centrifuged for 24hrs at 36000rpm at 4°C in SW41 Ti swing out rotor. 1ml fractions were collected using the Brandel density gradient fractionation system with upward displacement. Proteins were TCA precipitated, pellets washed 3x with cold acetone resuspended in 100 μL of 2xSDS Laemmli buffer, boiled for 5min and samples were analyzed by western blotting. On a separate experiment samples were spiked with aldolase (158kDa) and thyroglobulin (690kDa) as migration markers for the fractionation.

Purification of Recombinant His_6-NEDD8 Constructs

Critical

All *in vitro* experiments were performed with freshly produced/purified His_6-NEDD8 proteins each time, with no prior freezing. Constructs were expressed in BL21 codon plus bacteria grown and induced at 37°C for 2hrs with 0.5mM IPTG and lysed immediately in 50mM Tris-HCl pH7.5, 0.5M NaCl, 10% Glycerol, 10mM Imidazole, 0.2% Tween-20 and 5mM β-mercaptoethanol at 4°C for 30min then sonicated (3x 45% amplitude, 30 s) and centrifuged at 20000 g for 30min at 4°C. Supernatant was incubated with 300 μL of pre-washed nickel beads for at least 4hrs at 4°C. Beads were extensively washed in lysis buffer and finally washed in 50mM Tris-HCl pH7.5, 150mM NaCl, 5mM β-mercaptoethanol. Material was then eluted in 20mM Tris-HCl pH7.5, 150mM NaCl, 0.5M Imidazole, 5mM β-mercaptoethanol. After analysis by PAGE followed by Coomassie Blue staining, fractions were dialysed in 20mM Tris-HCl pH7.5, 150mM NaCl, 0.5mM DTT and stored at 4°C for subsequent *in vitro* analysis.

Purification of Recombinant GST HSP70 Constructs

Constructs were expressed in BL21 codon plus bacteria grown and induced at 37°C for 2hrs with 0.5mM IPTG. Cells were harvested and lysed in 50mM Tris-HCl pH7.5, 50mM NaCl, 0.5mM DTT and sonicated (amplitude 50%, 3 bursts of 30 s). After 20000 g centrifugation supernatant was incubated with 100 μL of pre-washed GST beads for at least 4hrs at 4°C. Beads were then extensively washed with 50mM Tris-HCl pH7.5, 150mM NaCl, 0.1% Triton X-100 with a final wash in 50mM Tris-HCl pH7.5, 150mM NaCl, 0.5mM DTT. Sequential elutions were performed with 50mM Glutathione pH7.5, 50mM Tris-HCl pH7.5, 150mM NaCl, 0.5mM DTT. Eluted fractions were analyzed by SDS-PAGE stained with Quick Coomassie and best fractions were pooled and dialysed against 50mM Tris-HCl pH7.5, 150mM NaCl, 0.5mM DTT. Extracts were finally cleared by 30000 g centrifugation.

Measurements of HSP70 ATPase Activity

Bacterially expressed His_6-HSP70 ATPase domain (residue 1-402, unless otherwise indicated) was prepared (final concentration 2 μM) in assay buffer (100mM Tris-HCl pH7.5, 20mM KCl, 6mM $MgCl_2$) in 50 μL final volume. This construct contains the linker domain which stimulates the ATPase activity of HSP70 (Jiang et al., 2007). BSA, NEDD8, Ubiquitin or HSP40 were added at the indicated concentration and incubated at 4°C for 2hrs to allow binding. The reaction was started by adding 0.5 μL of 20mM ATP. After 2hrs of incubation at 37°C, 25 μL of the reaction mixture were added to 75 μL of green malachite reagent (SIGMA, ATPase activity assay kit) on a 96-well plate and incubated for 30min at room temperature. The absorbance was determined at 620nm with a Polarstar Omega apparatus (BMG). To correct for non-enzymatic hydrolysis of ATP, the absorbance of a sample lacking HSP70 but otherwise treated identically was subtracted.

Isolation of Poly-NEDD8 Conjugates from Cell Extracts and ATPase Activity Measurement

U2OS cell lines stably expressing His_6-NEDD8 and deleted for NEDP1 were incubated in lysis buffer (20mM Tris-HCl pH7.5, 0.5M NaCl, 10mM Imidazole, 0.2% Tween-20, 5mM β-mercaptoethanol) at 4°C for 30min then sonicated (30% amplitude, 5sec burst) and centrifuged at 20000 g for 30min at 4°C. Supernatant was incubated with 50 μL of pre-washed nickel beads for at least 4hrs at 4°C. Beads were extensively washed in high salt washing buffer (20mM Tris-HCl pH7.5, 1M NaCl, 10mM Imidazole, 0.2% Tween 20, 5mM β-mercaptoethanol) and finally washed three times with washing buffer without Tween-20. Material was then eluted in elution buffer (20mM Tris-HCl pH7.5, 0.5M NaCl, 500mM Imidazole, 5mM β-mercaptoethanol) twice in 100 μL then dialysed against 100mM Tris-HCl pH7.5, 20mM KCl, 6mM $MgCl_2$ at least 10000 fold. For subsequent ATPase activity measurements, 20 μL were incubated with or without 500ng of bacterially expressed His_6-NEDP1 at 37°C for 2hrs in 100 μL final of 100mM Tris-HCl pH7.5, 20mM KCl, 6mM $MgCl_2$. Reactions were then transferred at 4°C and 2 μM of HSP70 ATPase domain (1-402) were added and incubated for 2hrs to allow binding. Reactions were started by adding ATP at 200 μM final concentration and incubated at 37°C for 2hrs. ATPase activity was measured as described above.

In Vitro Binding Assay

100nM of bacterially expressed GST or various GST-HSP70 constructs were incubated with 100nM of bacterially expressed His_6-NEDD8, His_6-HSP40 in 300 μL binding buffer (50mM Tris-HCl pH7.5, 150mM NaCl, NP-40 1%, 1mM DTT) for 1hr at 4°C then 20 μL of GST beads were added to each reaction for 1hr at 4°C under gentle rotation. Beads were washed 3x with binding buffer and 50 μL of SDS loading buffer was added before boiling. Supernatants were analyzed by western blotting. In the experiment in Figure S7A, ATP was included at 4mM.

In Vitro NEDDylation Assay

50 μL reactions were set up including recombinant proteins: 300ng GST-NAE, 5 μg Ubc12 and 10 μg His_6-NEDD8 in 50mM Tris-HCl, pH 7.5, 2mM ATP, 5mM $MgCl_2$, 2mM DTT. Reactions were incubated at 37°C for 5hrs. His_6-NEDD8 conjugates were isolated with 10 μL of pre-washed Ni^{+2}-agarose beads in 500 μL of 50mM Tris-HCl, pH 7.5, 0.5M NaCl for 2hrs at room temperature. Beads were extensively washed with Tris-HCl, pH 7.5, 0.5M NaCl before elution (2x100 μl) in Tris-HCl, pH 7.5, 0.5M NaCl, 250mM imidazole. Dialysis and ATPase activity measurements were performed as with the isolation of poly-NEDD8 conjugates from cell extracts (see above).

GST Pull-Down from Cell Extracts

Harvested cells were incubated in binding buffer (100mM Tris-HCl pH7.5, 20mM KCl, 6mM $MgCl_2$) at 4°C for 30min and sonicated (30% amplitude, 5sec burst) then centrifuged at 20000 g for 30min at 4°C. Supernatant was incubated with 10 μg of GST or GST-HSP70 constructs at 4°C at least 2hrs. Then 50 μL of pre-cleared (binding buffer supplemented with 20 μg of molecular grade BSA) GST beads were added and incubated for 1hr under gentle rotation. Beads were washed 3x with binding buffer supplemented with 0.5% NP-40 and 50 μL of SDS loading buffer was added before boiling. Eluates were analyzed by western blotting.

QUANTIFICATION AND STATISTICAL ANALYSIS

Image Gauge (western blot), Leica LAS (FRAP analysis), SEQUEST 3G and the SORCERER 2 (mass spectrometry analysis) were used. Statistical details including the statistical tests used can be found in figure legends. Values represent the average ± SEM. P values were calculated using two-tailed unpaired Student's t test, n values represent the number of independent experiments or number of animals used, as indicated in figure legends.

DATA AND CODE AVAILABILITY

The SILAC proteomics data generated during this study are available at MassIVE in the following link: ftp://massive.ucsd.edu/MSV000084214/.

Cell Reports, Volume *29*

Supplemental Information

The Balance between Mono- and NEDD8-Chains

Controlled by NEDP1 upon DNA Damage Is

a Regulatory Module of the HSP70 ATPase Activity

Aymeric P. Bailly, Aurelien Perrin, Marina Serrano-Macia, Chantal Maghames, Orsolya Leidecker, Helene Trauchessec, M.L. Martinez-Chantar, Anton Gartner, and Dimitris P. Xirodimas

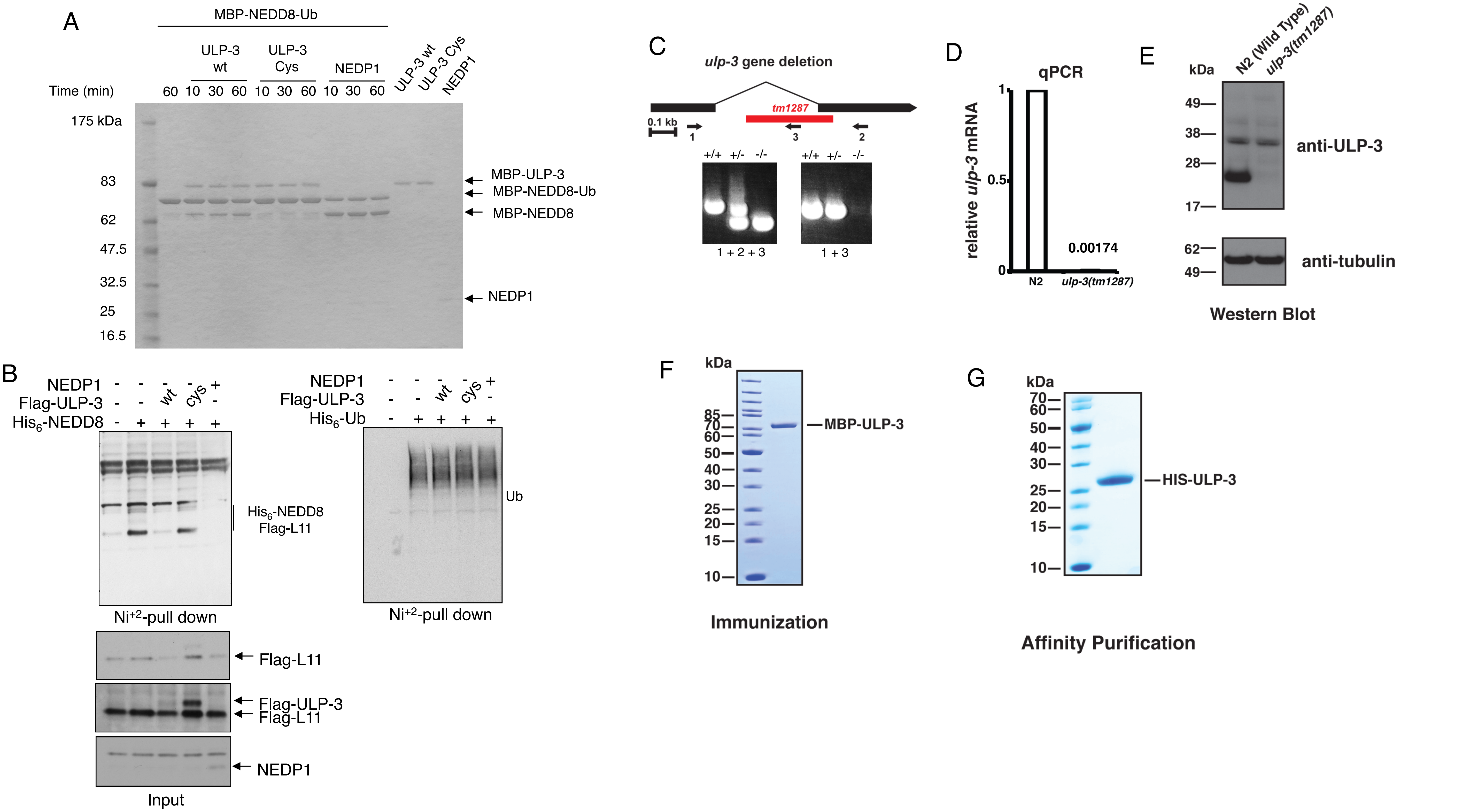

Figure S1. ULP-3 has NEDD8 processing and de-NEDDylating activity. The *ulp-3(tm1287)* allele abolishes *ulp-3* gene expression, related to Figure 1.

(A) Bacterially expressed wild type MBP-ULP-3 or Cys catalytic mutant were tested *in vitro* for NEDD8 processing activity using the MBP-NEDD8-Ub fusion. As control wild type bacterially expressed NEDP1 was used.

(B) H1299 cells were transfected with the indicated constructs. In all samples Flag-L11 was also co-transfected. 48hrs post-transfection His_6-NEDD8 conjugates were isolated with Nickel beads and western blot analysis was performed with anti-Flag antibody. Total cell extracts (input) were analysed with the indicated antibodies. Similar experiments were performed using His_6-ubiquitin and after Nickel purification ubiquitin conjugates were detected with anti-ubiquitin antibody (right panel).

(C) The *tm1287* allele structure demonstrated by the PCR products generated by the indicated primers. +/+, +/- and -/- indicate the N2/N2, N2/*ulp-3* and *ulp-3/ulp-3* genotypes respectively.

(D) The gene disruption is demonstrated by the *ulp-3* mRNA level measured by real time PCR. Relative expression levels were determined using γ-tubulin as standard (*tbg-1*). Fold induction was calculated as previously described (Bailly et al., 2010).

(E) Wild type and *ulp-3(tm1287)* mutant worms were synchronised and grown under standard conditions. Protein extraction was performed as described in Methods and the ULP-3 protein levels analysed by western blotting. α-tubulin was used as protein loading control.

(F) Recombinant MBP-ULP-3 protein, used for de-NEDDylation *in vitro* assays and rabbit immunisation (Supplemental information Fig. 1A), was produced in BL21 cells and purified with amylose resin as described in Methods. Purified MBP-ULP-3 protein was analysed by SDS-PAGE followed by Coomassie Blue staining.

(G)Recombinant His-ULP-3 (used to purify ULP-3 antibodies) was produced in BL21 cells and purified on a Cobalt column then analysed by SDS-PAGE followed by Coomassie Blue staining.

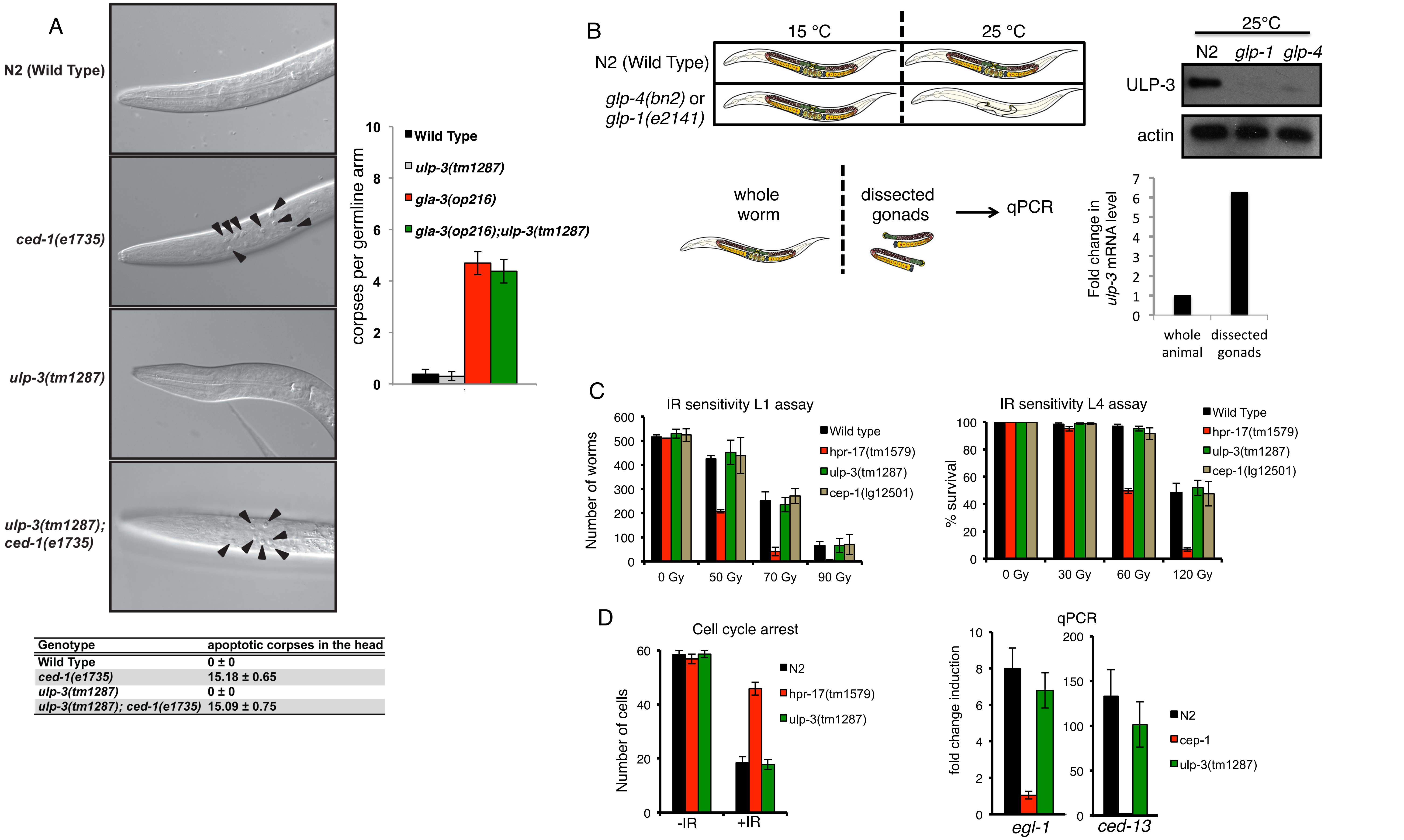

Genotype	apoptotic corpses in the head
Wild Type	0 ± 0
ced-1(e1735)	15.18 ± 0.65
ulp-3(tm1287)	0 ± 0
ulp-3(tm1287); ced-1(e1735)	15.09 ± 0.75

Figure S2. ULP-3 specifically affects the DNA damage-induced apoptotic pathway, related to Figure 1.

(A) Left panel: the apoptotic corpses that persist in the head until the L1 larvae stage in *ced-1(e1735)* animals were scored as in Bailly et al., 2010. Representative DIC pictures of the indicated genotype are shown. Average number of corpses, +/- SEM is represented (*n*=15) in the lower panel. In contrast to wild type, the *gla-3(op216)* mutant displays an elevated number of germ cell apoptosis without DNA damage referred as physiological apoptosis level. The double mutant *ulp-3(tm1287); gla-3(op216)* does not show a significant decrease in the number of apoptotic cells suggesting that ULP-3 does not regulate physiological germ cell apoptosis (right panel).

(B) Thermo-sensitive mutants glp-4(bn2ts) do not develop germ line tissue into adult gonads when exposed to restrictive temperature at early development stage, leading to animals essentially devoid of germ cell as depicted in the upper panel. Wild type and glp-4(bn2ts) synchronised worms were both switched to restrictive temperature at the L1 larvae stage (25°C). After reaching young adult stage, worm protein were extracted and ULP-3 protein level analysed by western blotting (upper right panel). RNA from whole animals or from dissected gonads from wild type animals was used for qPCR to monitor *ulp-3* expression. Values were normalised to tubulin expression and presented as fold change relative to whole animal *ulp-3* expression (lower right panel).

(C) The *ulp-3* deletion allele does not increase irradiation sensitivity following L1 (upper graph) or L4 (middle graph) larvae stage irradiation. Assays were performed as described (Bailly et al., 2010) using *hpr17* deletion mutant as a positive control (the RAD17 worm homologue).

(D) Left panel: *ulp-3* deletion does not impact on the mitotic cell cycle arrest triggered by the DNA damage checkpoint signalling pathway in *C. elegans* germ cells. Arrested cells were monitored and counted as previously described (Bailly et al., 2010). Right panel: Expression levels of 2 p53 regulated genes (egl-1 and ced-13) after IR (90Gy) in different genetic backgrounds. γ-tubulin expression was used to normalise values. Data represent the average (*n*=3) +/- SEM.

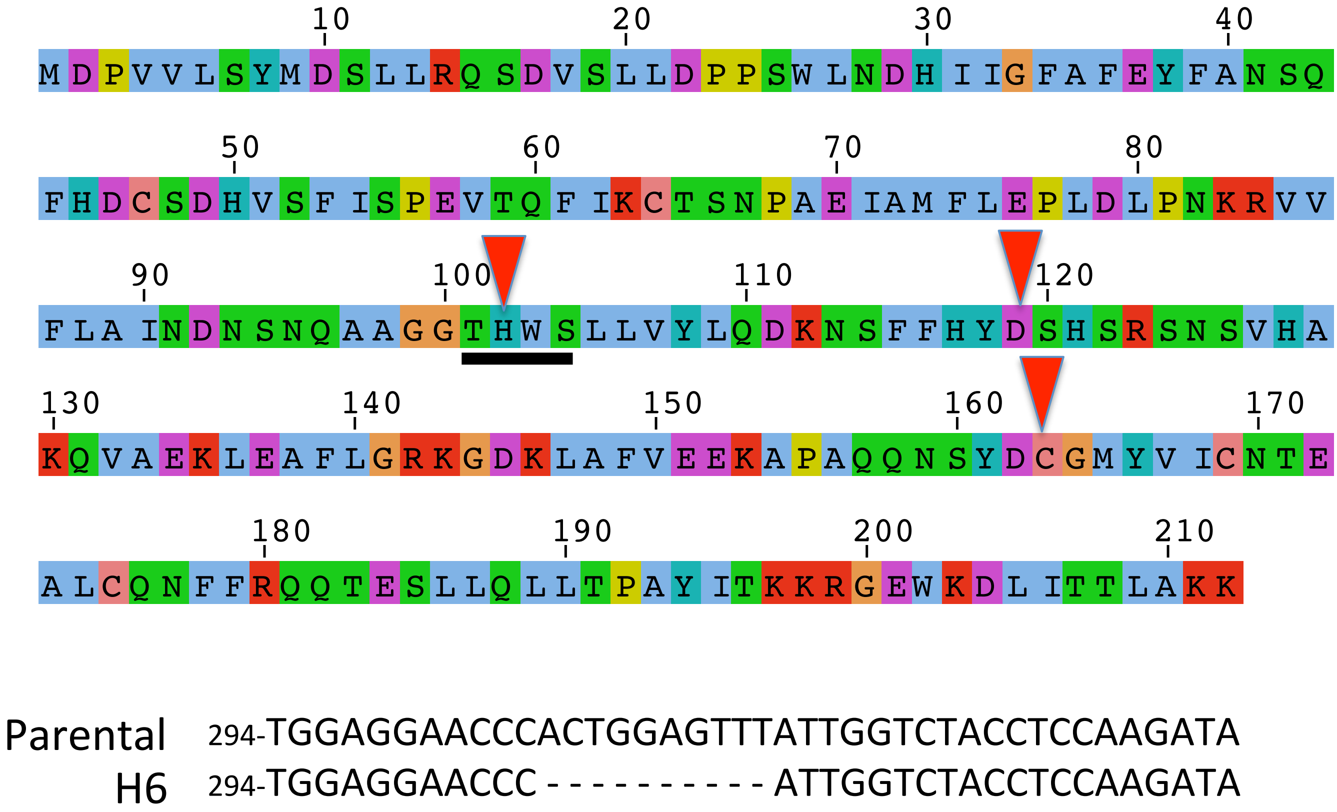

Figure S3. NEDP1 deletion by CRISPR/Cas9, related to Figure 3.
Sequencing of NEDP1 PCR products using genomic DNA as template from parental or H6 U2OS cells confirmed the indicated deletion of 10 base pairs in the NEDP1 gene creating an out of frame sequence and a stop codon 90 bases downstream. Red arrows indicate the position of the catalytic triad in NEDP1 and the black line the target area of the guide sequence at amino acid level.

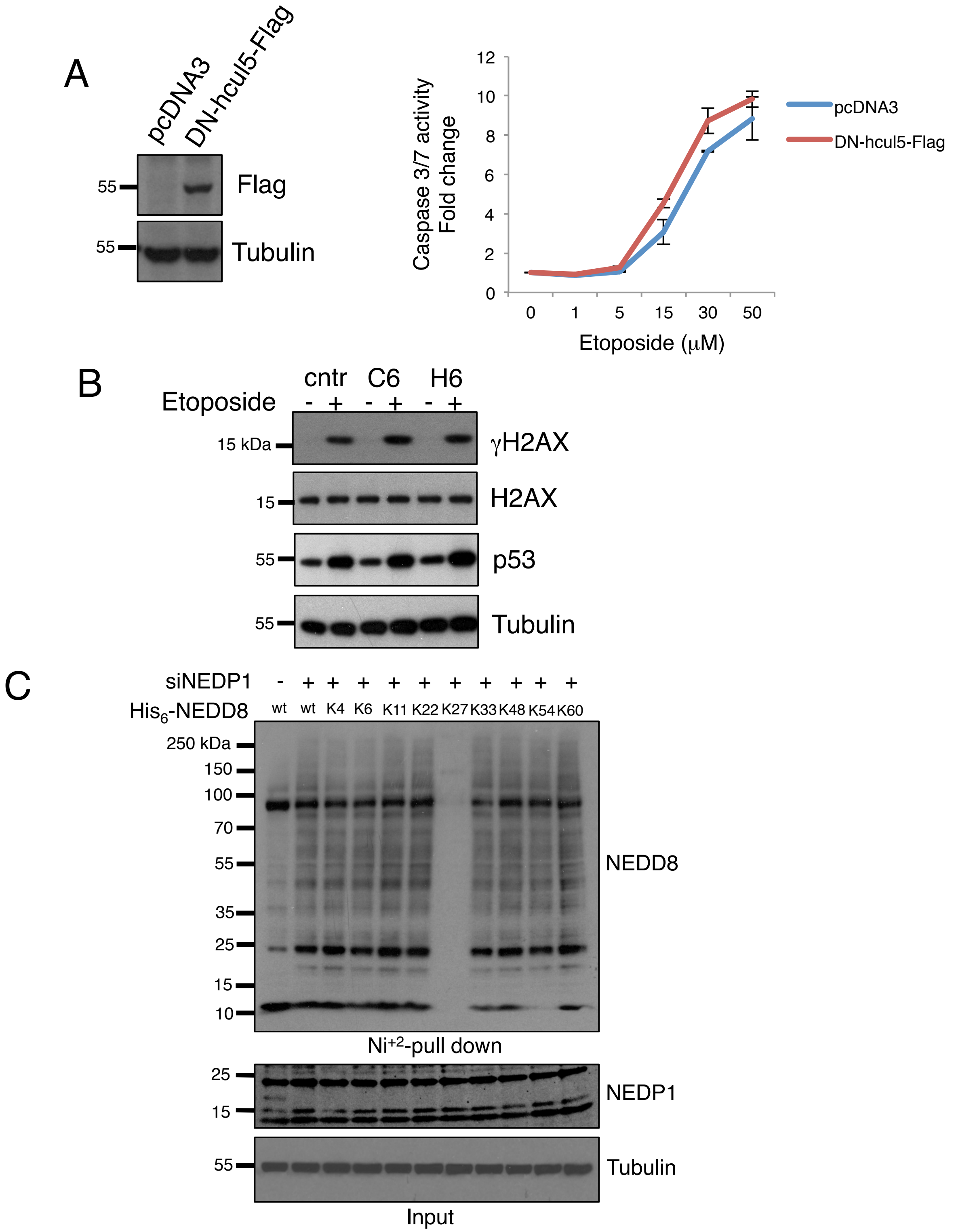

Figure S4. Characterisation of the DSBs-induced apoptosis in NEDP1 knockout cells and the modification of KR NEDD8 mutants, related to Figures 3, 4.

(A) U2OS cells transfected with pcDNA3 or the dominant negative cullin 5-Flag expression construct were seeded in 24 well plates and treated with etoposide as indicated for 15hrs before measurement of caspase 3/7 activity as before. Graph represents average values (*n*=2) +/- SEM.

(B) Parental or NEDP1 knockout U2OS cells (clones C6, H6) were treated with etoposide (50μM for 15hrs) and cell extracts were analysed with the indicated antibodies by western blotting.

(C) U2OS cells transfected as described in Methods with the indicated siRNAs, wild type and KR NEDD8 expression constructs, were used for nickel pull-downs. Eluates and total cell extracts were used for western blotting.

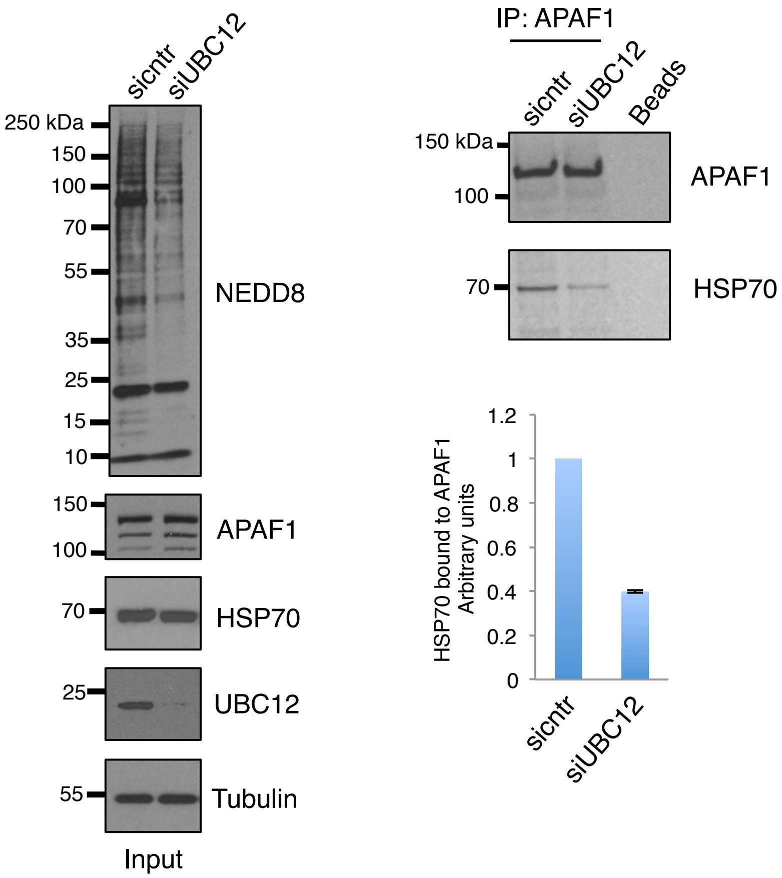

Figure S5. UBC12 knockout decreases the APAF1-HSP70 binding in H6 cells, related to Figure 5.
NEDP1 knockout U2OS cells (H6) were transfected either with control or UBC12 siRNAs. Extracts were used for APAF1 immunoprecipitations (IP) and western blotting as described in Fig. 5D. Extracts from the sicntr cells were used for the beads only IP. The HSP70 signal in the IPs was quantified and the average values (*n*=2) +/- SEM relative to the control is presented in the bottom right graph.

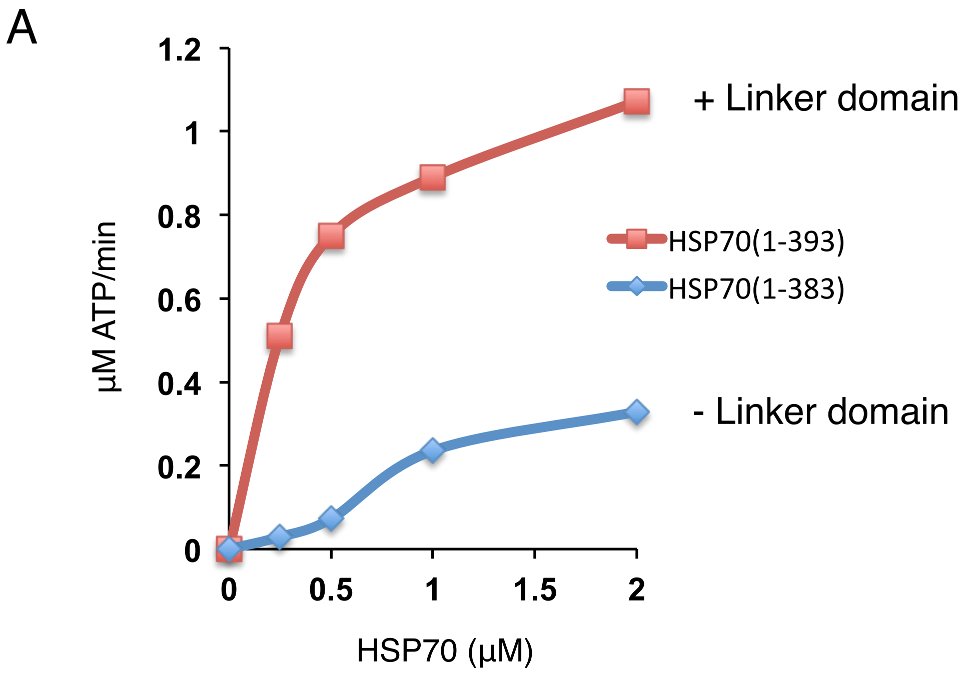

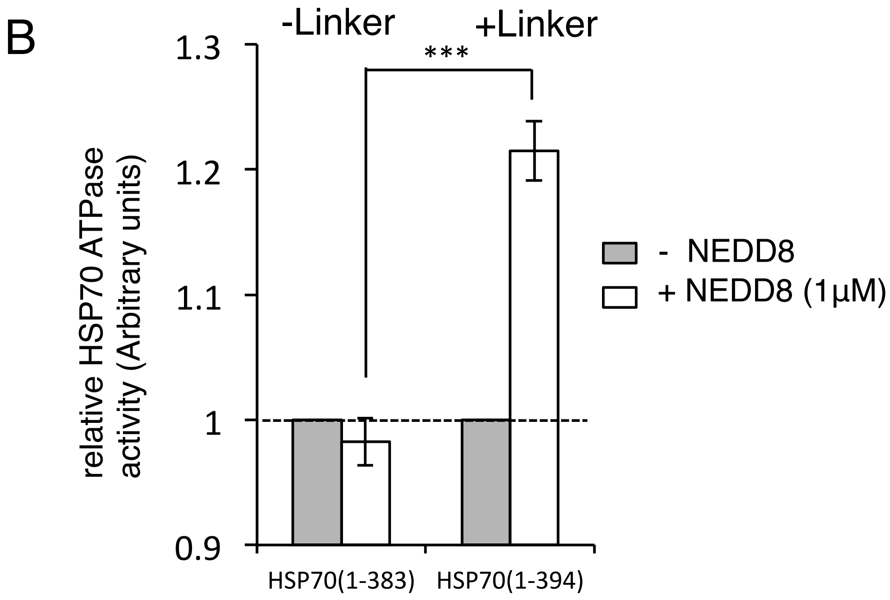

Figure S6. The Linker domain is critical for the stimulation of the HSP70 ATPase activity by NEDD8, related to Figure 6.

(A) The ATP hydrolysis activity of the HSP70 ATPase domain in the presence or absence of the Linker domain (383-393) was measured.

(B) The effect of NEDD8 on the HSP70 ATP hydrolysis activity in the presence or absence of the linker domain in HSP70. Average values (n=5) +/- SEM with p value <0.001.

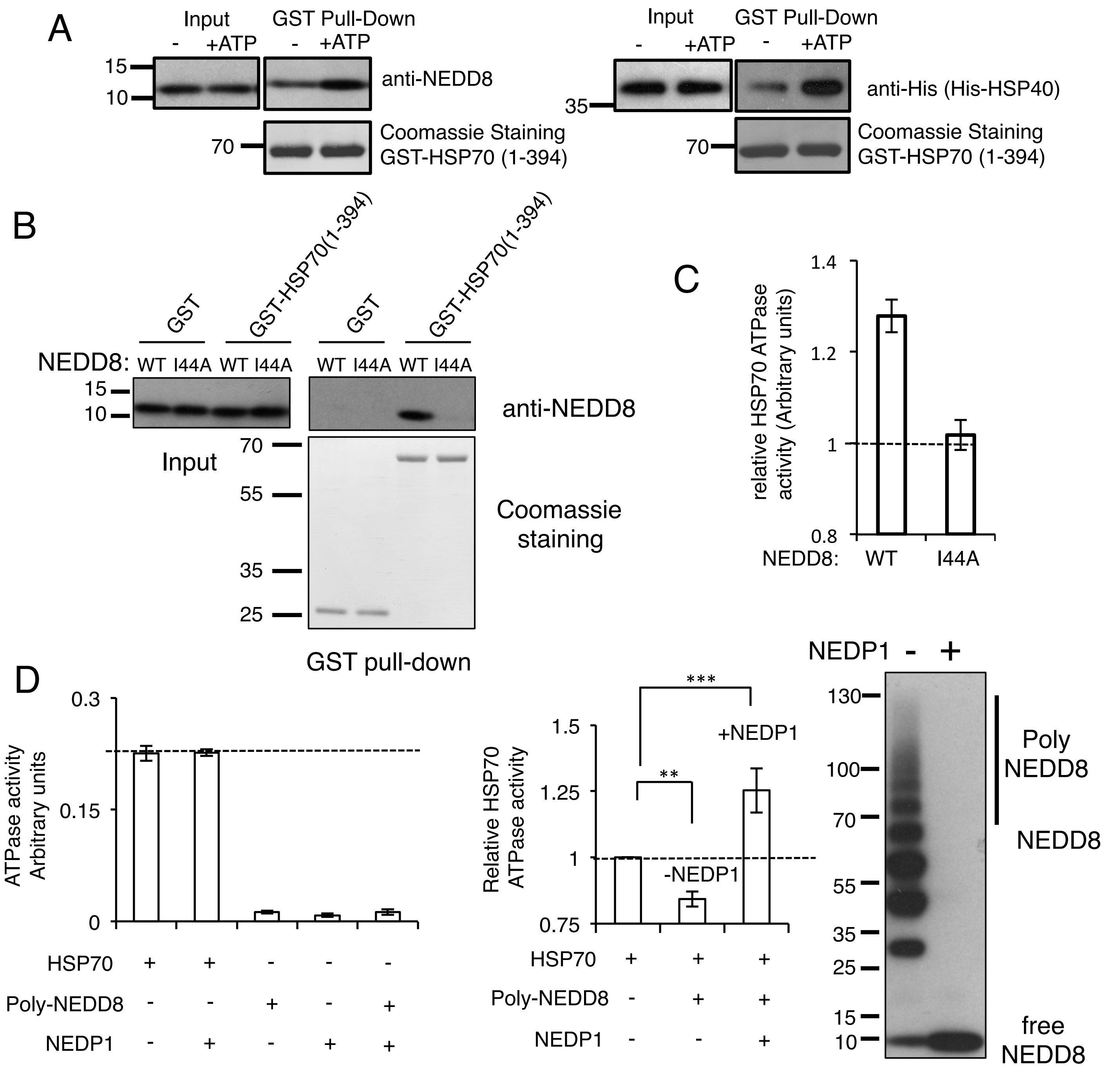

Figure S7. Characterisation of the effect of NEDD8 on HSP70 ATPase activity, related to Figure 6.

(A) ATP stimulates the HSP70 interaction with NEDD8 (left panel) or HSP40 (right panel) in GST-HSP70 pull-downs.

(B) The NEDD8 point mutant I44A fails to bind the HSP70 ATPase domain *in vitro* in a GST pull-down assay.

(C) The I44A mutation inhibits the NEDD8 stimulatory effect on HSP70 ATPase activity *in vitro*. Average values (*n*=5) +/- SEM with p value <0.001.

(D) Poly-NEDD8 conjugates synthesized *in vitro* using recombinant His_6-NEDD8 were isolated by Ni^{2+} pull-down and subjected to NEDP1 digestion (right panel) and processed as in Fig.6F. (*n*=5 +/- SEM, p values **≤ 0.01, ***≤ 0.001).
